# A computational study on effect of *Cymbopogon citratu* and *Juniperus virginiana* against *Spodoptera litura*

**DOI:** 10.1038/s41598-025-93785-w

**Published:** 2025-05-14

**Authors:** Jyotsna Bandi, Viswajith Mulpuru, Jalaja Naravula

**Affiliations:** Department of Biotechnology, Vignan’s Foundation for Science Technology and Research (Deemed to be University), Vadlamudi, Andhra Pradesh 522213 India

**Keywords:** *Spodoptera litura*, Pest management, Essential oils, Lemongrass, Cedarwood, Molecular docking, Sustainable agriculture, Pest control, Plant-based pesticides, Biotechnology, Computational biology and bioinformatics, Plant sciences

## Abstract

*Spodoptera litura*, commonly known as the leaf cutworm, is a destructive agricultural pest that poses significant threats to crop yields. In light of the growing resistance to conventional chemical insecticides, this study investigates the potential of lemongrass (*Cymbopogon citratus*) and cedarwood (*Juniperus virginiana*) essential oils as sustainable, eco-friendly alternatives for controlling *S. litura*. Using in-silico techniques, including homology modeling, protein structure validation, and protein-ligand docking, the binding affinities of lemongrass and cedarwood bioactive compounds against critical proteins in *S. litura*, such as cytochrome c oxidase, alpha-glucosidase, octopamine receptors, and chemosensory protein has been evaluated. The results showed that the compounds: chamazulene, robustoflavone, cynaroside, hinkoflavone, spathulenol, robustaflavone, and amentoflavone exhibited strong inhibitory potential, with superior binding stability and interaction energies compared to the chemical insecticide chlorpyrifos. Additionally, synergistic effects were observed when combining compounds from lemongrass and cedarwood, which improved binding stability and enhanced multi-target inhibition. This study highlights the promise of these natural compounds as viable, environmentally friendly pest control agents and provides a foundation for developing plant-based bioinsecticides to manage *S. litura*. Future experimental research is needed to validate these findings in field applications and assess their broader ecological impacts.

## Introduction

*Spodoptera litura*, commonly known as the leaf cutworm, is a significant agricultural pest belonging to the order *Lepidoptera* and family *Noctuidae*^1,2^. This species is extensively distributed across South and East Asia, as well as Oceania, thriving primarily in frost-free tropical and subtropical climates and expanding to cooler regions during summer^1,3^. Notably, *S. litura* is a highly polyphagous pest, feeding on over 40 plant families, including economically essential crops from families such as *Brassicaceae, Cucurbitaceae, Fabaceae, Malvaceae, Poaceae,* and *Solanaceae*^1,4,5^. While the larvae primarily feed on plant leaves, at higher densities, they can cause severe defoliation, consuming multiple plant parts, which results in considerable crop damage^6,7^. A field experiment conducted in Jabalpur, Madhya Pradesh, examined the damage caused by *S. litura* to cabbage crops during the 2017–2018 rabi season. The infestation began in mid-November, with 0.1 larvae per head leading to 2% crop damage, and reached a peak in mid-January with 3.17 larvae per head and a 54% head infestation rate^8^. Chemical insecticides, including carbamates (e.g., methomyl), pyrethroids (e.g., fenpropathrin), and organophosphates (e.g., chlorpyrifos), have been used in an attempt to control *S. litura* populations^9^. However, repeated and extensive insecticide application has led to widespread resistance in *S. litura* across various chemical classes, posing a serious challenge for pest management on a global scale^10^. In light of these challenges, integrated pest management (IPM) strategies have emerged as promising eco-friendly alternatives. IPM approaches utilize a diverse toolkit including biological control agents, microbial treatments, cultural practices, and plant resistance to sustainably control *S. litura* populations while reducing reliance on chemical pesticides^11^. This paradigm shift is mirrored in efforts to manage other *Spodoptera* species, such as *Spodoptera frugiperda*, whose global spread has sparked investigations into sustainable management strategies that include botanical, chemical, biological, and pheromonal controls^12^. In this regard, botanical pesticides like *Azadirachta indica* and *Eucalyptus globulus* show potential as eco-friendly alternatives to conventional insecticides, especially in field applications where they offer pest control efficacy with a reduced environmental impact^13^. Azadirachtin, for example, disrupts key biological processes in *S. litura*, affecting protein expression and development through mechanisms such as inhibition of the ecdysone receptor^14^.

Other plant-derived compounds have shown potential against *Spodoptera* pests, offering eco-friendly pest control solutions. Essential oils from *Lippia sidoides* and thymol, for instance, have been effective against *S. frugiperda* while being more selective and less harmful to beneficial predators, such as *Podisus nigrispinus*, compared to chemical insecticides like deltamethrin^15^. In Africa, where *the S. frugiperda* invasion threatens maize yields and food security, studies have identified 69 plant-derived pesticides as viable, sustainable options, particularly for small-scale farmers^16^. Sustainable alternatives, including herbal pesticides and nano-formulations, have also shown effectiveness against *S. litura*, presenting environmentally friendly approaches to overcome pesticide resistance and protect non-target organisms^17^. Moreover, specific compounds demonstrate promising selective toxicity against *Spodoptera* species. For example, methyl benzoate has shown high efficacy against *S. frugiperda* with minimal impact on non-target species and natural predators^18^. Similarly, myrrh extract, used alone or in combination with insecticides like profenofos or chlorfluazuron, has been effective in controlling *Spodoptera littoralis* larvae in cotton, demonstrating both standalone and complementary pest control capabilities^19^. Furthermore, extracts from *Solidago graminifolia*, particularly those with quercetin, exhibit strong insecticidal activity against *S. frugiperda* larvae, likely due to the compound’s binding affinity for acetylcholinesterase, a critical enzyme in pest physiology^20^. Research on the interactions between sex pheromones and odorant-binding proteins, such as SfruGOBP2 in *S. frugiperda*, reveals potential target genes for environmentally friendly pest control through mating disruption^21^. In the present study, an in-silico analysis was conducted to evaluate the effects of compounds from lemongrass (*Cymbopogon citratus*) and cedarwood (*Juniperus virginiana*) on multiple protein targets of *S. litura*, including cytochrome c oxidase subunit 1 (COX1) (A0A895KRZ4), alpha-glucosidase (AGI) (A0A9J7DWA6), octopamine receptor (ORs) (A0A9J7EV60), carboxylic ester hydrolase (CEHs) (A0A9J7DZU6), and chemosensory protein (CSPs) (7E8L), are critical targets for pest regulation due to their essential roles in the insect’s physiology and behavior. COX1 is vital for energy production in the respiratory chain, and its inhibition can impair the insect’s fitness. AGI is crucial for carbohydrate metabolism, so targeting it can hinder the insect’s energy acquisition, affecting growth and reproduction. The ORs regulate movement and feeding; thus, its disruption can reduce these activities significantly. CEHs assist in detoxifying harmful compounds, and their inhibition can increase the insect’s susceptibility to natural insecticides. Lastly, the CSPs is essential for detecting environmental cues; impairing its function can hinder food and mate location, impacting reproductive success. Collectively, these proteins provide strategic avenues for developing innovative, eco-friendly pest management solutions that reduce reliance on traditional chemical insecticides and promote sustainable agricultural practices. By identifying potential interactions with these molecular targets, this study aims to explore alternative pest control options that could contribute to the development of novel, sustainable management strategies against *S. litura*.

## Material and methods

### Homology modeling and protein preparation

The *S. litura* proteins: COX1, AGI, ORs**,** and CEHs were retrieved from the UniPort database, and CSPs was retrieved from the PDB database. CSPs and COX1 3D PDB structures are available in the PDB (https://www.rcsb.org/) and UniPort databases (https://www.uniprot.org/). For the remaining proteins: CEHs, AGI, and ORs, 3D PDB structures were modelled using SWISS MODEL. Protein sequences of CEHs, AGI, and ORs were obtained from the UniPort database and submitted to the SWISS-MODEL server (https://swissmodel.expasy.org/), a popular tool for automated comparative protein modeling. It predicted the Alpha fold modeling technique^22^. This method is based on the assumption that proteins with similar sequences also have similar structures, allowing a model to be built using a homologous protein’s known structure. The modeled structures were submitted to SAVESv6.0 for quality check. The quality of SWISS-modelled CEHs, AGI, and ORs proteins were analyzed using SAVESv6.0- Structure validation server (https://saves.mbi.ucla.edu/). It uses a variety of parameters to evaluate model quality, including ERRAT to access overall model quality, reflecting accuracy^23^, PROCHECK for stereochemical quality assessment, and VERIFY 3D to evaluate the model’s compatibility with its amino acid sequences^24^. This validation ensures a comprehensive evaluation of the predicted model’s accuracy and reliability, providing a solid foundation for subsequent functional and structural analyses. After validation,** e**nergy minimization was performed on the modeled protein structures of CEHs, AGI, and ORs using the online energy minimization server YASARA^25^ (https://www.yasara.org/). The energy-minimized protein PDB structures were retrieved by submitting the YASARA energy minimization scene file (.sec) to the YSARA view. Now, the energy-minimized proteins were subjected to find the functional domains using InterPro. The functional domains of COX1**,** AGI, ORs, CEHs, and CSPs were predicted using InterPro^26^. InterPro (https://www.ebi.ac.uk/interpro/) is a diverse protein signature database that predicts domains, homologous superfamily, and functional motifs within a protein sequence. Finally, Active sites were predicted for the proteins:COX1**,** AGI, ORs, CEHs, and CSPs using PrankWeb for performing docking studies. PrankWeb^27^ (https://prankweb.cz/) is a machine learning-based method to predict ligand binding sites of proteins.

### Plant essential oil components as ligands

Lemongrass -*Cymbopogon Citratu*^28,29^ and Cedarwood- *Juniperus Virginiana*^30^, Hammam et al.,^31^ are the plants selected for the study because of their repellent and insecticidal properties. The compounds of these plants were collected from the IMPPAT (https://cb.imsc.res.in/imppat/home) database. The compounds were downloaded in 3D PDBQT from the IMPPAT database. The selection of ligand compounds for docking studies from lemongrass and cedarwood showcases a diverse array of phytochemicals that could potentially exhibit significant bioactivity against *S. litura* proteins. Lemongrass contains a total of 57 compounds, including notable constituents such as bicyclogermacrene, chamazulene, and citral, which are known for their insecticidal and repellent properties. In contrast, cedarwood features 9 compounds, with key components like hinokiflavone, robustaflavone, and amentoflavone. The presence of a wide variety of phytochemicals in both plants not only enhances the likelihood of finding effective ligands but also allows for the exploration of synergistic effects among combined compounds, paving the way for the development of potent bioinsecticides targeting multiple proteins involved in the pest’s physiological processes. This diverse chemical profile positions both lemongrass and cedarwood as promising sources for identifying lead compounds in the fight against agricultural pests. Chlorpyrifos, a widely used commercially available organophosphate pesticide, has been selected as the reference ligand in this study due to its established efficacy in controlling *S. litura*, a major agricultural pest that damages crop such as cotton, tobacco, and soybean. Chlorpyrifos exerts its insecticidal action by inhibiting acetylcholinesterase (AChE) in the insect’s nervous system, leading to paralysis and death^32^. However, the widespread and prolonged use of chlorpyrifos has raised concerns regarding the development of insect resistance, environmental toxicity, and risks to non-target organisms, including human health^33^. Given these concerns, there is an urgent need to explore natural, eco-friendly alternatives with comparable or superior insecticidal properties. In this study, chlorpyrifos serves as a benchmark for molecular docking analyses^34^, allowing for a comparative evaluation of the binding affinities of natural compounds derived from lemongrass and cedarwood, with key proteins- COX1**,** AGI, ORs, CEHs and CSPs in *S. litura*. These studies aim to identify natural, eco-friendly alternatives that can match or surpass chlorpyrifos in effectiveness, offering safer pest control solutions.

### Protein-ligand docking

Before performing protein-ligand docking, both protein and ligand molecules were prepared in PDBQT format. The ligand molecules were directly downloaded in 3D-PDBQT format from the IMPPAT database. For the proteins, PDB structures were converted into PDBQT format using AutoDockTools-1.5.7^35^. The modeled 3D PDB structures of the receptor proteins: COX1**,** AGI, ORs, CEHs, and CSPs were prepared using AutoDockTools-1.5.7. This preparation involved the addition of hydrogens (all hydrogens and merge non-polar), assignment of Kollman charges, removal of water molecules, and the addition of AD4 atom types, after which the structures were saved in PDBQT format. The Kollman charges assigned to the proteins were as follows: −6.0 for COX1, −4.0 for the AGI, 12.0 for the ORs, -6.339 for CEHs, and -4.0 for the CSPs. After converting the protein PDB structures into PDBQT format, grid boxes were created for each receptor protein to define the search space for docking. For CEHs, the grid box was centered at coordinates x = 4.937, y =  −4.093, and z = 0.272, with dimensions x = 94, y = 102, and z = 78, for AGI the coordinates were centered at x = 10.5, y = -0.729 and z = -10.179, with dimensions x = 82, y = 84 and z = 94, for ORs the coordinates were centered at x = 4.819, y = 6.923 and z = −15.934, with dimensions x = 58, y = 70 and z = 84 , for COX1 was centered at x =  −19.945, y =  −2.027, and z = 11.826, with dimensions x = 60, y = 46, and z = 54 and for the CSPs was centered at x = 0.605, y =  −3.395, and z = 19.636, with dimensions x = 46, y = 50, and z = 48. These grid boxes defined the regions within which docking was performed, ensuring the accurate identification of ligand binding sites on each receptor protein. With the grid parameters of each protein, a configuration file was created to perform AutoDock Vina. Docking studies were performed using AutoDock Vina 1.2.0^36^ to explore the interactions between several insect proteins: COX1, AGI, ORs, CEHs, and CEHs and plant-derived ligands from lemongrass and cedarwood. The best binding affinities of the ligands interacting with each protein were identified and consolidated for further grouped docking analysis. Next, the top-scoring ligands from each plant were grouped to perform a group docking study using AutoDock-Vina-GPU-2.1^37^, targeting the same set of proteins. This group docking aimed to assess potential synergistic interactions between ligands from both plants on the insect proteins. By combining the top ligands, we aimed to demonstrate a synergistic mode of action, enhancing the efficacy of these compounds against insect targets. The interactions of the grouped ligands with each protein were analyzed and compared to identify key interacting residues within the proteins. Visualization of these interactions was carried out using LigPlot + v.2.2 software^38^, allowing us to map the binding interactions and hydrogen bonds formed between the ligands and the protein active sites. This analysis provided insights into the potential enhanced activity of the ligand combinations.

### Molecular dynamics simulations (MDS)

Molecular dynamics simulations (MDS) were conducted on the PDB-docked structures of the highest-scoring grouped docked ligands with their respective proteins to assess the stability of ligand-protein interactions. The simulations were performed using GROMACS 2024.2^39^ with the CHARMM36 all-atom force field^40^. To initiate the simulations in GROMACS, topology files for both the protein and ligand were created. The protein topology was generated directly in GROMACS using the CHARMM36 force field. However, since GROMACS lacks a specific force field for ligand topology generation, Swiss Param ^41^ was used to create the ligand topology. These individual topology files were then combined to generate a comprehensive topology file for the protein-ligand complex, which was further processed to prepare it for MDS. The subsequent steps included solvation, minimization, and equilibration, all performed using the CHARMM36 all-atom force field. Solvation of the protein–ligand complex was achieved using the linear constraint solver (LINCS) algorithm, with the addition of water molecules based on the TIP3P explicit water model to generate covalent bonds within the particles. The system was then neutralized by balancing Cl^−^ and Na^+^ ions, followed by energy minimization (EM) using the steepest descent algorithm until the maximum force was reduced to below 2.39 kcal/mol. Particle mesh Ewald (PME) was employed for evaluating electrostatic interactions. For equilibration, the system was coupled using the Berendsen thermostat and pressure coupling to achieve a stable temperature of 300 K and pressure of 1 bar under NVT and NPT ensembles, respectively. The final MDS was executed with a 2-fs integration time step over 100 ns at constant temperature and pressure (300 K and 1 bar) using the leapfrog algorithm. The molecular dynamic trajectories were analyzed using GROMACS utilities to estimate and determine the protein-ligand interactions. Root mean square deviation (RMSD) calculations were performed on the protein, as well as on the best-scored ligand, using a chemical compound as a reference to evaluate the stability of the protein-ligand complex. The rigidity of the secondary structure was assessed by calculating the root mean square fluctuation (RMSF) for the ligand in the MDS complex. Additionally, the strength of the protein-ligand interactions was determined by analyzing energy variances throughout the simulation time. For structural alignment and visualization, LigPlot software^38^ was employed. The results of the MDS were graphically represented using GraphPad Prism^42^.

## Results and discussion

### Homology modeling and quality assessment

**Fig. 1:**
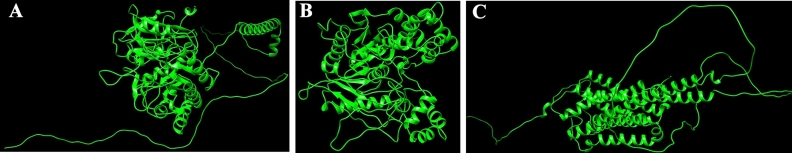
(**A**–**C**) SWISS Modelled structures of proteins AGI, CEHs, and Ors. (**A**) SWISS modeled PDB structure of AGI from Chimera, (**B**) SWISS modeled PDB structure of CEHs from Chimera, (**C**) SWISS modeled PDB structure of ORs from Chimera.

**Fig. 2:**
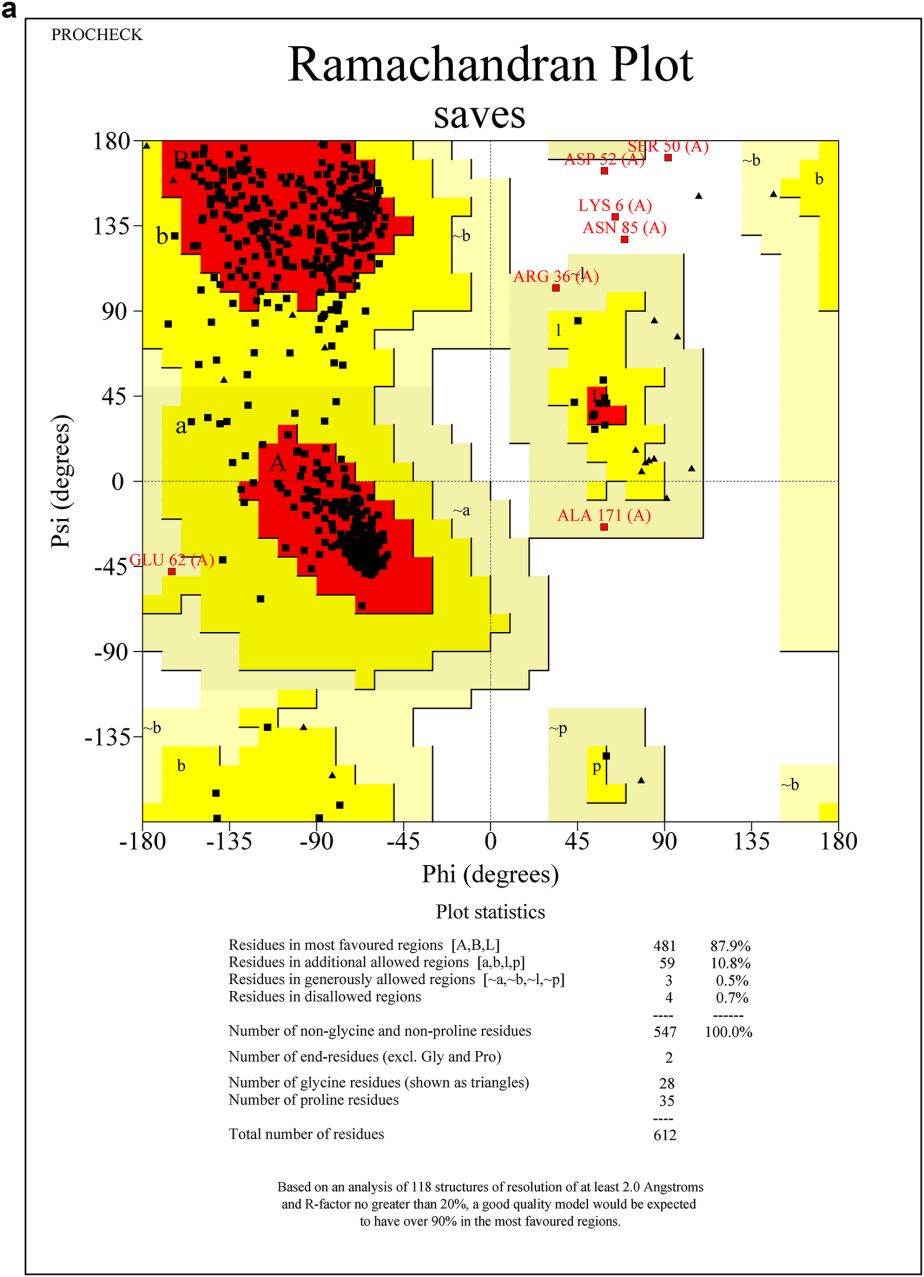

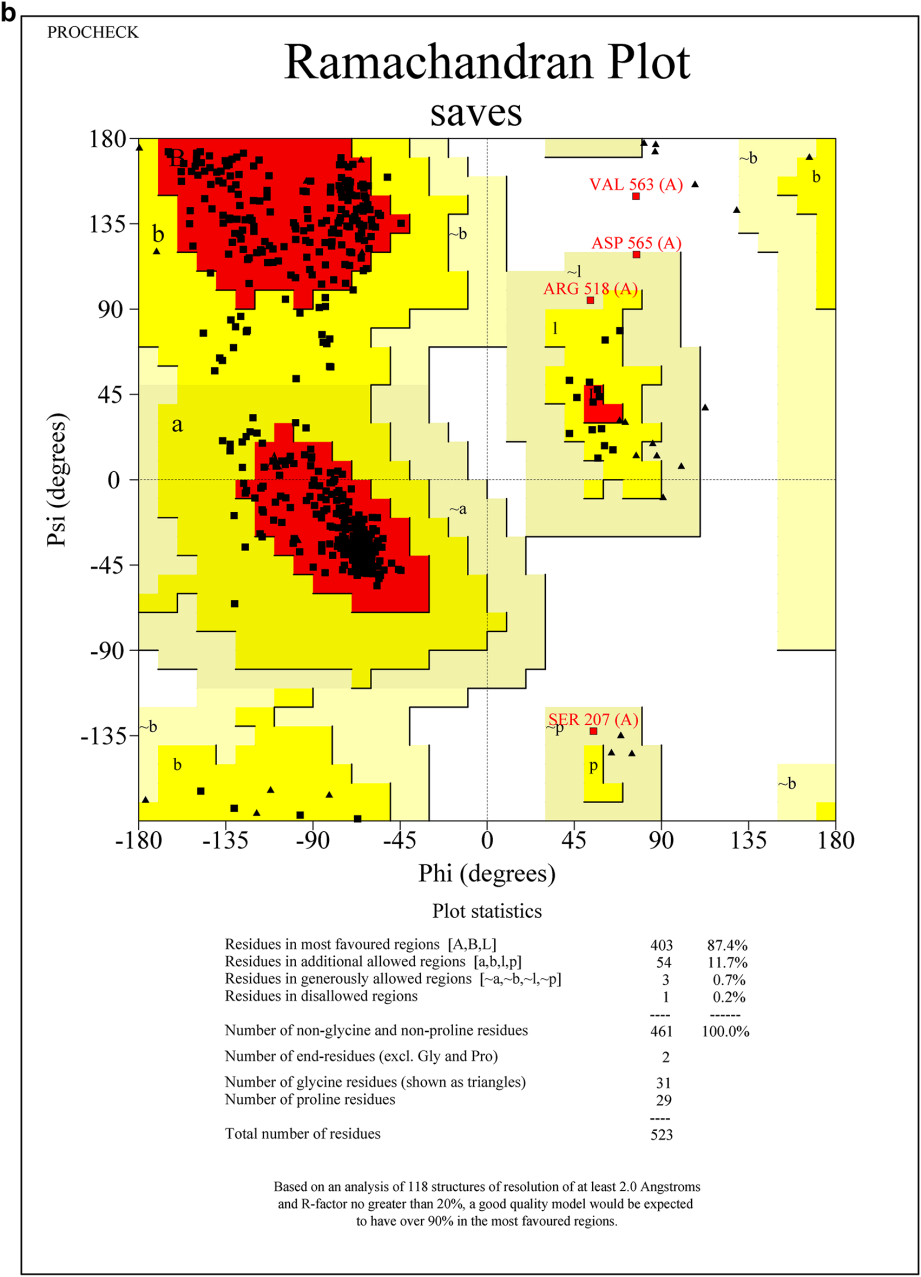

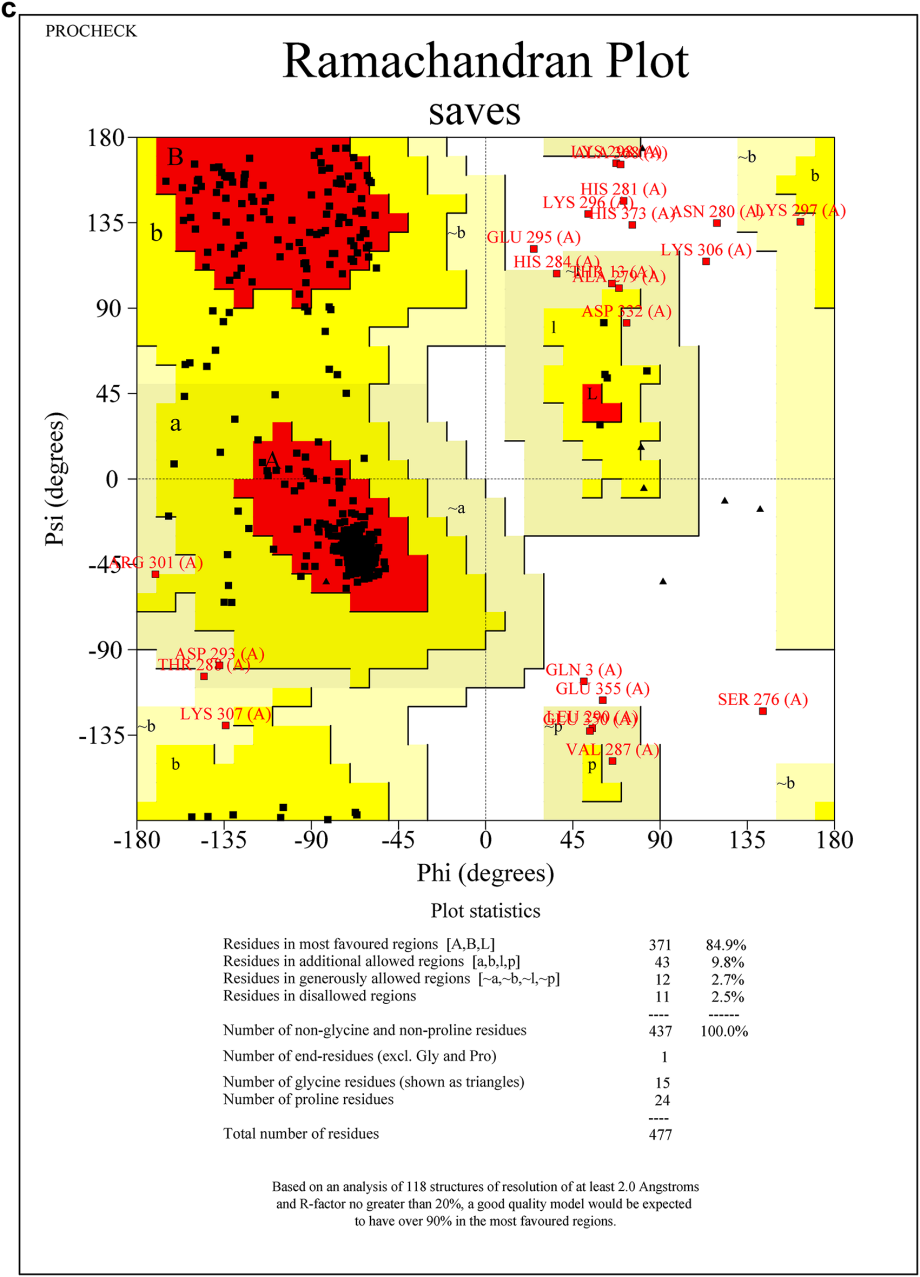
(**A**–**C**) The quality of the modeled protein structures- AGI, CEHs, and ORs was validated using the SAVES v6.0 structure validation server through Ramachandran plot analysis. (**A**) Ramachandran plot analysis of SWISS modeled AGI PDB structure from SAVES v6.0 structure validation server, (**B**) Ramachandran plot analysis of SWISS modeled CEHs PDB structure from SAVES v6.0 structure validation server, (**C**) Ramachandran plot analysis of SWISS modeled ORs PDB structure from SAVES v6.0 structure validation server.

Protein sequences for CEHs, AGI, and ORs were modeled using the SWISS-MODEL server. The 3D PDB models are represented in (Fig. 1A–C). For CEHs, the AlphaFold DB model of A0A2H1VAI9_SPOFR, based on the *Spodoptera frugiperda* (Fall Armyworm) sequence (Gene: SFRICE041916.2), was selected as the template, exhibiting 89.87% sequence identity. The AGI model was built using the AlphaFold DB model of A0A7E5VBV1_TRINI from *Trichoplusia ni* (Cabbage Looper) (Gene: LOC113492491), with a 70.10% sequence identity. For the ORs, the AlphaFold DB model of OAR_HELVI from *Heliothis virescens* (Tobacco Budworm Moth) (Gene: OAR_HELVI) was used, showing 96.22% sequence identity. The quality of the modeled protein structures was validated using the SAVES v6.0 Structure Validation Server. Ramachandran plot analysis was performed to assess the backbone dihedral angles (phi and psi) of the residues across all three models. The analysis revealed that a high proportion of residues in each model occupy the most favored regions of the plot, with 87.4% for the AGI model (Fig. 2A), 84.9% for the CEHs model (Fig. 2B), and 85.5% for the ORs model (Fig. 2C). Additional residues were located in allowed regions, with a minimal percentage found in generously allowed and disallowed regions (ranging from 0.2 to 2.5%). Outliers such as Valine 563 in the AGI model and Histidine 281 in the CEHs model were noted but represent isolated cases that do not significantly impact the overall structural stability. Glycine and proline residues were positioned as expected, further supporting the structural integrity of the models. Overall, the structures are considered reliable, with only minor refinements required for optimization. These models are well-suited for subsequent computational analyses, including molecular dynamics simulations and docking experiments.

### Functional domain, energy minimization, and active site prediction

**Fig. 3:**
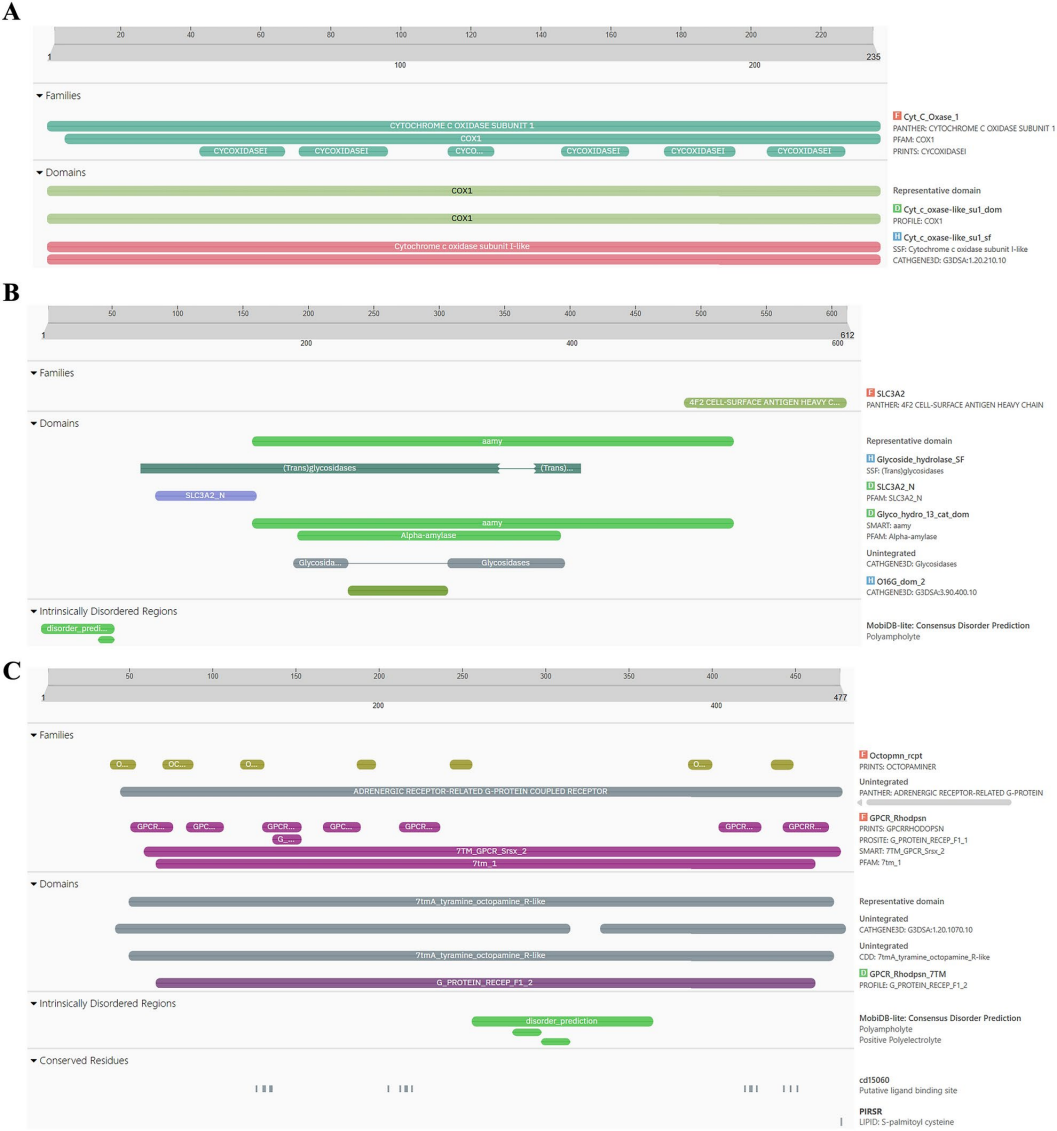

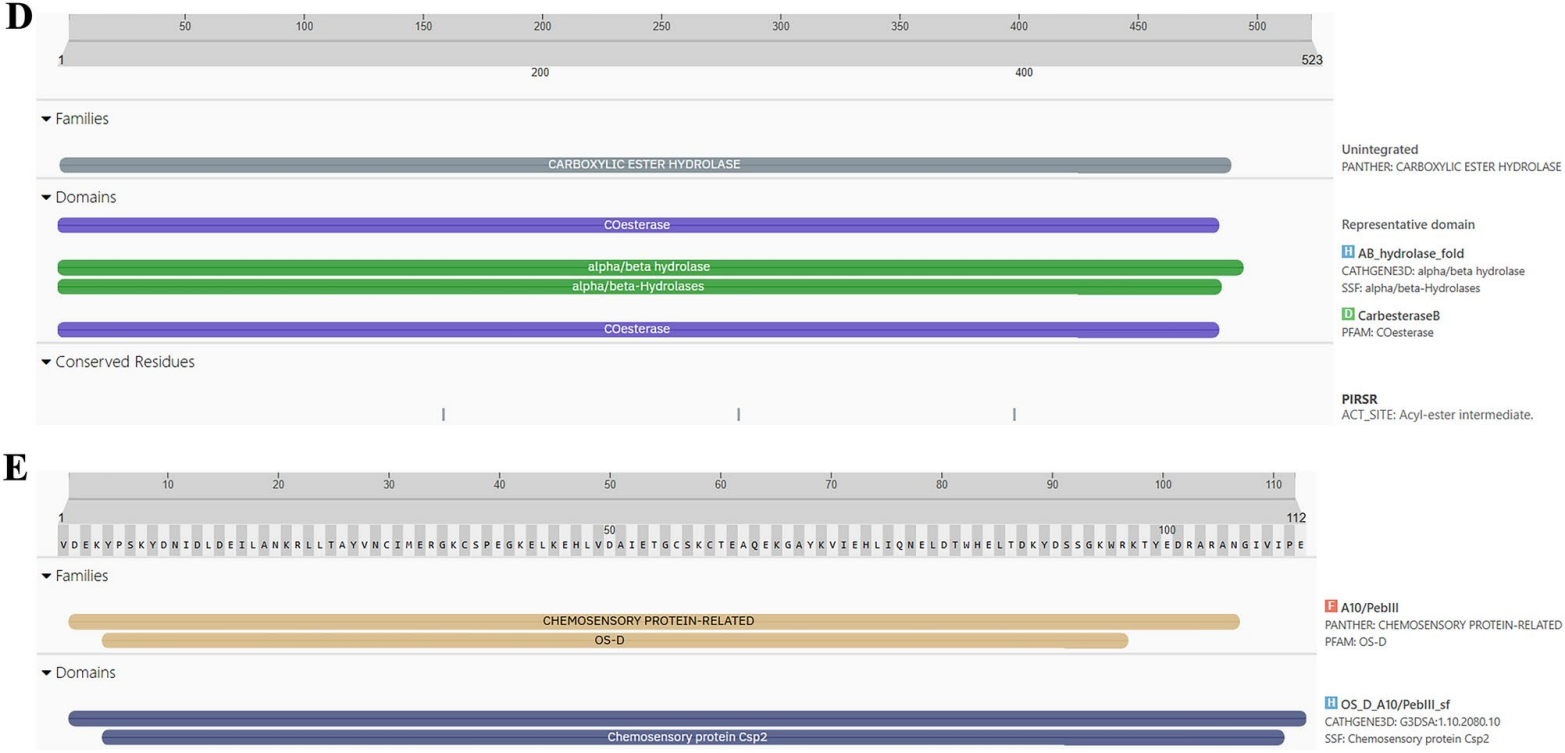
(**A**–**E**) Functional domains, homologous superfamily, and functional motifs within a protein sequence analysis of proteins- COX1, AGI, ORs**,** CEHs, and CSPs through InterPro. (**A**) Functional domain analysis of COX1 sequence through InterPro, (**B**) Functional domain analysis of AGI sequence through InterPro, (**C**) Functional domain analysis of ORs sequence through InterPro, (**D**) Functional domain analysis of CEHs sequence through InterPro, (**E**) Functional domain analysis of CSPs sequence through InterPro.

The proteins:COX1**,** AGI, ORs, CEHs, and CSPs were analyzed using InterPro to identify domains, homologous superfamilies, and functional motifs within their sequences. The functional domains, homologous superfamily, and functional motifs within a protein sequence analysis of proteins- COX1**,** AGI, Ors, CEHs, and CSPs through InterPro were represented in (Fig. 3A–E). The proteins exhibit remarkable structural and functional diversity, each playing a pivotal role in critical biological processes. COX1 (Fig. 3A) is essential for cellular respiration, specifically within the electron transport chain. The COX1 domain in this protein drives energy production through its involvement in proton pumping and electron transfer. Its transmembrane and cytoplasmic regions support efficient energy transduction, facilitating electron flow and contributing to the generation of a proton gradient necessary for ATP synthesis. This makes COX1 indispensable for cellular energy metabolism, underscoring its integral role in oxidative phosphorylation^43^. AGI (Fig. 3B) is a key enzyme in carbohydrate metabolism, containing domains from the Glyco_hydro_13_cat_dom and SLC3A2 families. These domains enable the enzyme to hydrolyze starch and sugars by breaking glycosidic bonds, essential for the conversion of complex carbohydrates into glucose. Its transmembrane and cytoplasmic regions suggest its membrane-bound nature, positioning it to facilitate not only carbohydrate processing but also the transport of sugars across cellular membranes, which is critical for cellular energy production and glucose homeostasis^44^. ORs protein (Fig. 3C) belongs to the GPCR (G-protein coupled receptor) superfamily, specifically within the ORs family. Characterized by the 7tmA, tyramine, and GPCR rhodopsin-like domains, this receptor mediates signal transduction across the cell membrane. The seven transmembrane helices facilitate the relay of external signals to intracellular signaling pathways, regulating critical physiological processes such as hormone response and neurotransmission. This makes the ORs central to modulating behaviors related to stress, locomotion, and hormonal balance^45^. CEHs (Fig. 3D) feature a CO esterase domain and a Carboxylesterase type B domain, which place it within the Alpha/Beta hydrolase fold superfamily. These domains indicate its function as a carboxylesterase enzyme, hydrolyzing ester bonds in xenobiotics and endogenous compounds. Its conserved serine active site is vital for catalysis, making it a key player in detoxification and metabolism. The presence of both cytoplasmic and non-cytoplasmic domains suggests the enzyme operates across various cellular environments, coordinating detoxification processes and metabolic regulation^46^. CSPs (Fig. 3E), part of the Insect odorant-binding protein family and Csp2 superfamily, are involved in detecting odorants and pheromones critical for insect sensory biology. This protein plays a central role in chemical signal detection and transduction, which is vital for behaviors like foraging, mating, and navigation. Recognizing environmental signals, enables organisms to respond to their surroundings, emphasizing its role in sensory adaptation and behavioral responses. Together, these proteins illustrate the sophisticated evolution of domain architecture and functional specificity, supporting vital processes like hydrolytic activity, energy production, sensory signal transduction, and carbohydrate metabolism. Their structural complexity ensures the maintenance of cellular and physiological homeostasis across different organisms, highlighting the intricate mechanisms underlying life’s essential functions^47^.

Energy minimization is a crucial step in refining protein structures for molecular dynamics simulations, as it helps to resolve steric clashes and optimize atomic positions, leading to more stable and accurate models. Using YASARA, significant energy reductions were observed in several key proteins: COX1(−91,162.2 to −117,351 kJ/mol), AGI (−250,203.2 to −337,654.7 kJ/mol), Ors (−184,864.7 to −254,877.7 kJ/mol), CEHs (−212,819.6 to −279,727.0 kJ/mol), and CSPs (−56,539.6 to 74,956.7 kJ/mol). These results demonstrate that energy minimization effectively improves structural accuracy, making the proteins more suitable for downstream applications like molecular docking. Additionally, accurate active site prediction for each protein ensures that docking targets the correct binding regions, enhancing the reliability of the results. The Active site residues of the proteins- COX1**,** AGI, ORs, CEHs, and CSPs to perform docking studies were represented in (Table 1). Together, the above steps ensured more precise receptor-ligand interactions in further docking studies.

**Table 1:** Active site residues of the proteins- COX1**,** AGI, ORs, CEHs, and CSPs to perform docking studies.

Name of the protein	Active site sequences
COX1	A_157, A_160, A_161, A_170, A_171, A_173, A_178, A_181, A_182, A_185
AGI	A_164, A_166, A_191, A_192, A_193, A_194, A_195, A_196, A_225, A_227, A_230, A_231, A_232, A_235, A_275, A_276, A_295, A_298, A_299, A_331, A_332, A_404, A_406
CEHs	A_123, A_125, A_126, A_127, A_128, A_131, A_132, A_133, A_138, A_139, A_206, A_207, A_239, A_290, A_329, A_330, A_332, A_333, A_334, A_336, A_347, A_351, A_354, A_355, A_392, A_396, A_397, A_429, A_430, A_433, A_434, A_443, A_444, A_447, A_90
ORs	A_109, A_112, A_128, A_129, A_132, A_204, A_205, A_206, A_208, A_29, A_420, A_423, A_424, A_427, A_433, A_437, A_440, A_441, A_443, A_444
CSPs	A_10, A_11, A_43, A_47, A_60, A_62, A_63, A_66, A_8, A_9

### Protein-ligand docking

**Fig. 4:**
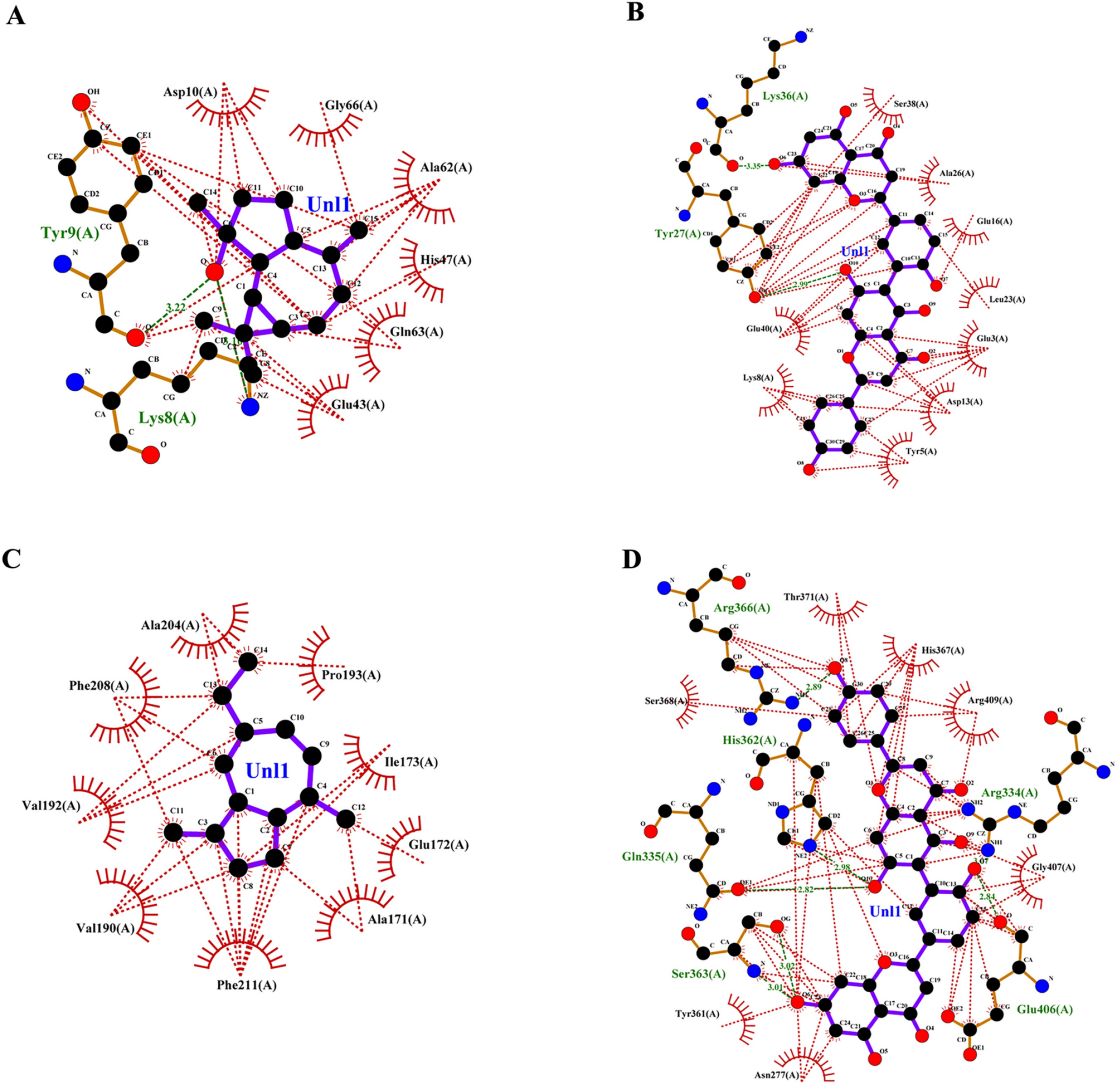

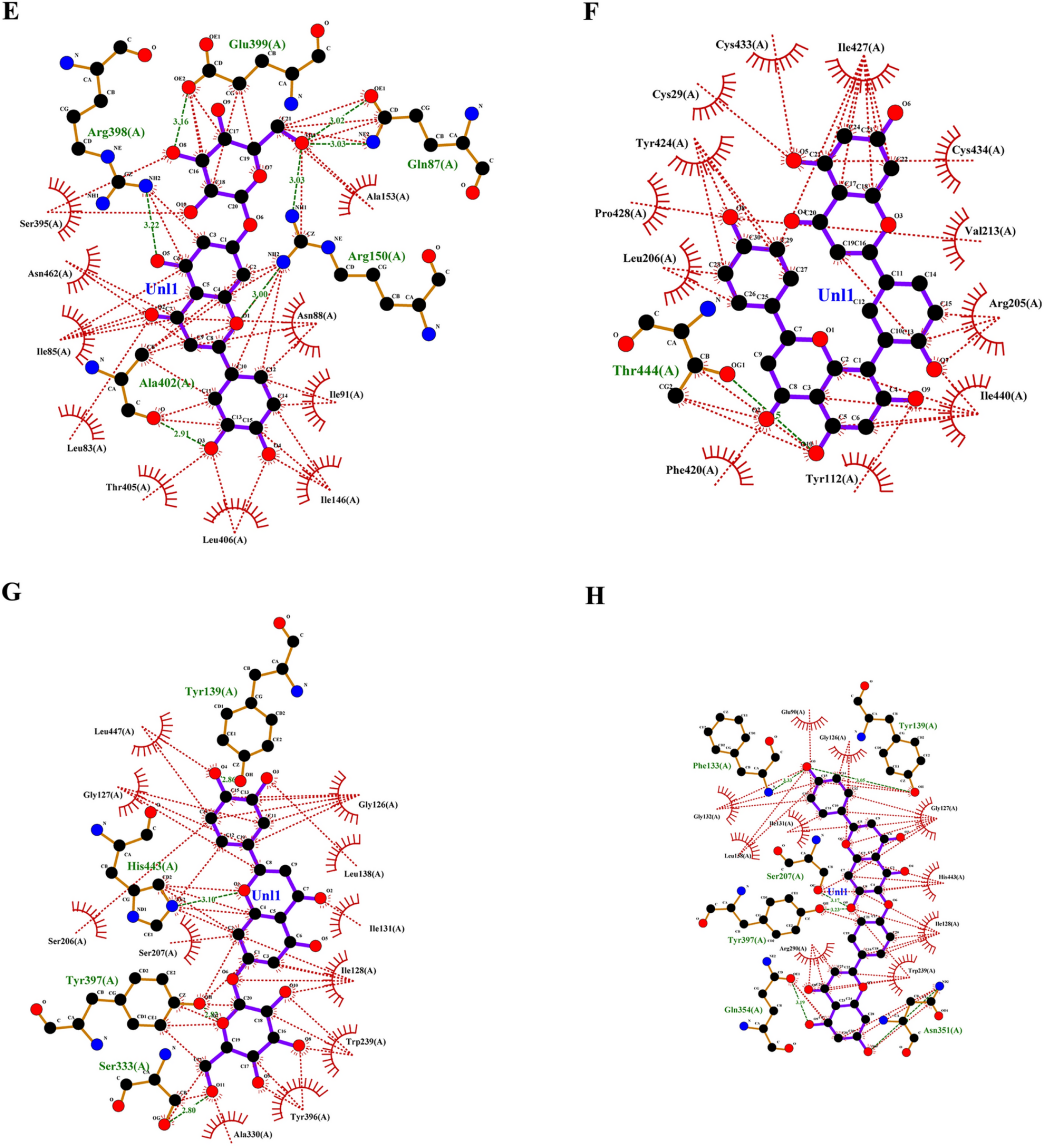

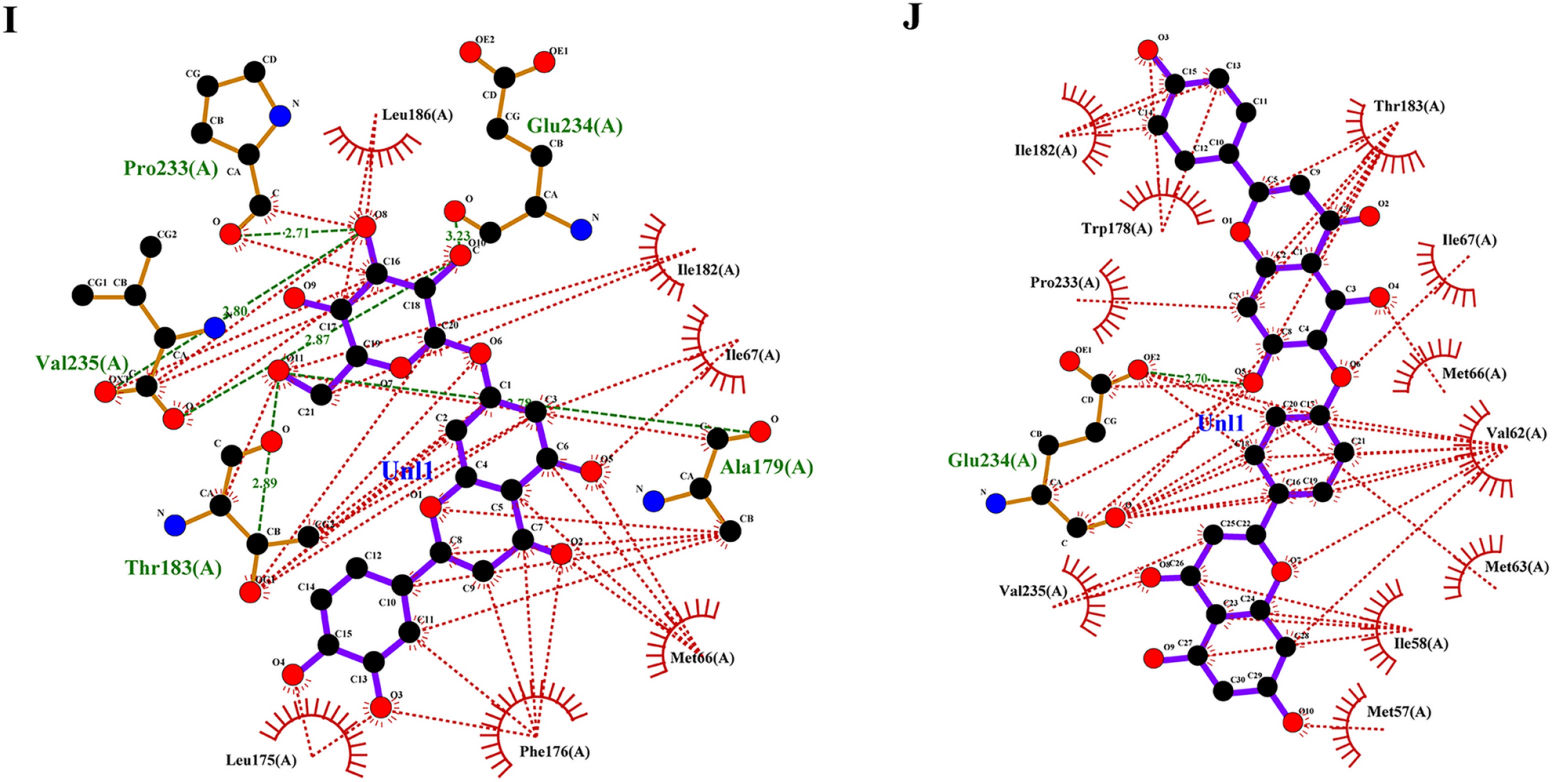
(**A**–**J**) The 2D interaction of best ligand compounds from lemongrass and cedarwood with *S. litura* proteins—COX1**,** AGI, ORs, CEHs, and CSPs using ligplot plus. (**A**) The 2D interaction of compound spathulenol from lemongrass with chemosensory protein of *S. litura*. (**B**) The 2D interaction of compound robustaflavone from cedarwood with chemosensory protein of *S. litura*. (**C**) The 2D interaction of compound chamazulene from lemongrass with AGI of *S. litura*. (**D**) The 2D interaction of compound robustaflavone from cedarwood with AGI of *S. litura*. (**E**) The 2D interaction of compound cynaroside from lemongrass with octopamine protein of *S. litura*. (**F**) The 2D interaction of compound amentoflavone from cedarwood with octopamine protein of *S. litura*. (**G**) The 2D interaction of compound cynaroside from lemongrass with CEHs of *S. litura*. (**H**) The 2D interaction of compound hinokiflavone from cedarwood with CEHs of *S. litura*. (**I**) The 2D interaction of compound cynaroside from lemongrass with cytochrome c oxidase of *S. litura*. (**J**) The 2D interaction of compound hinokiflavone from cedarwood with cytochrome c oxidase of *S. litura*.

Targeting invasive pest proteins with plant essential oils or bioactive compounds offers a sustainable approach to pest control. The process begins with selecting key pest proteins essential for survival or reproduction, such as enzymes or neural receptors. Bioactive compounds from essential oils were screened for their ability to bind and inhibit these proteins. Protein-ligand docking and molecular dynamics simulations help predict and validate the effectiveness of these interactions. This strategy can lead to the development of eco-friendly bioinsecticides that disrupt pest biology, reducing crop damage while being safe for the environment. From the docking studies, the binding affinities of various compounds of lemongrass (Table 2) with *S. litura* proteins- COX1, AGI, ORs, CEHs, and CSPs were observed. Among the tested compounds, cynaroside shows the highest binding affinity across most proteins, with a strong interaction of −37.656 kJ/mol for COX1, −37.2376 kJ/mol for CEHs, and −33.472 kJ/mol for both AGI and ORs, indicating its potent inhibitory potential. Similarly, chamazulene exhibits notable affinity for Ors (−35.9824 kJ/mol), making it another promising compound for targeting the ORs, a key modulator in insect nervous systems. Furthermore, spathulenol also shows a strong binding affinity towards COX1 (−34.3088 kJ/mol), indicating the potential for inhibiting essential metabolic pathways in *S. litura*. These compounds, particularly cynaroside, could be promising candidates for developing bioinsecticides targeting multiple physiological pathways in pests. From the cedarwood compounds docking studies (Table 3) with the same proteins mentioned above were observed, amentoflavone shows the strongest binding affinity with CEHs (−45.1872 kcal/mol) and COX1 (−44.7688 kcal/mol), indicating its potential as a potent inhibitor for these enzymes critical to insect metabolic processes. Robustaflavone also displays high binding affinity, particularly with AGI (−42.6768 kcal/mol) and CEHs (−41.0032 kcal/mol), suggesting its role in disrupting digestive and metabolic functions in *S. litura*. Hinokiflavone exhibits strong binding to CEHs (−44.7688 kcal/mol) and ORs (−40.5848 kcal/mol), making it a potential candidate for interfering with the insect’s nervous and metabolic systems. Additionally, cupressuflavone demonstrates considerable affinity for AGI (−38.0744 kcal/mol) and Ors (−40.1664 kcal/mol), further supporting its role as an effective inhibitor of these target sites. These compounds, especially amentoflavone and robustaflavone, show strong multi-target activity and are promising for the development of bioinsecticides against *S. litura*. The best ligand compounds from lemongrass and cedarwood essential oils based on their binding affinity with *S. litura* proteins—COX1**,** AGI, ORs, CEHs, and CSPs were mentioned in (Table 4). The 2D interaction profile of best ligand compounds from lemongrass and cedarwood with *S. litura* proteins—COX1**,** AGI, ORs, CEHs, and CSPs from the ligplot plus were represented in Fig. 4 (A, B, C, D, E, F, G, H, I & J). Docking interaction profile- residues of *S. litura* proteins—COX1**,** AGI, ORs, CEHs, and CSPs with consolidated best binding affinity ligand compounds from lemongrass and cedarwood; and also, with reference molecule chlorpyrifos were represented in (Table 5). From the analysis, lemongrass and cedarwood demonstrate a stronger and more diverse interaction profile with the analyzed proteins compared to chlorpyrifos, as evidenced by their ability to form a greater number of both hydrogen bonds and hydrophobic interactions. For instance, lemongrass forms hydrogen bonds with key residues in COX1 (GLU 234, ALA 179, THY 183), CEHs (TYR 139, HIS 443), and CSPs (TYR 9, LYS 8), suggesting its potential for stabilizing protein-ligand complexes through polar interactions. Additionally, cedarwood frequently exhibits hydrogen bonding with critical residues in proteins such as AGI (ARG 366, HIS 362), further enhancing its binding affinity. Hydrophobic interactions, essential for enhancing ligand binding in non-polar environments, are also more prominent for lemongrass and cedarwood across multiple proteins, such as their interactions with residues like ILE 67, MET 66, and PRO 233 in COX1 and LEU 206 and TYR 424 in ORs. In contrast, chlorpyrifos shows limited hydrogen bond interactions and forms fewer hydrophobic interactions with protein residues, which could limit its stability and affinity within the binding sites. This suggests that lemongrass and cedarwood, due to their richer interaction profiles, may exhibit better efficacy in modulating protein activity and could be more suitable candidates for bioactive or insecticidal applications compared to chlorpyrifos against *S. litura*.

**Table 2:** Binding affinity of ligands (compounds from lemon grass) interaction with *S. litura* proteins-—COX1**,** AGI, ORs, CEHs, and CSPs.

Name of the compound	Binding affinity in KJ/mol				
	CSPs	AGI	ORs	CEHs	COX1
Bicyclogermacrene	−22.5936	−25.5224	−30.5432	−30.5432	−22.5936
Bulnesol	−23.012	−32.6352	−33.472	−31.7984	−25.9408
Caffeic acid	−23.4304	−25.104	−26.7776	−25.9408	−20.5016
Camphene	−17.9912	−21.7568	−22.5936	−23.8488	−20.0832
Camphor	−18.828	−23.8488	−24.2672	−23.8488	−17.5728
Carotol	−23.4304	−28.8696	−30.1248	−29.7064	−21.7568
Carvacrol	−20.5016	−25.5224	−25.104	−26.3592	−22.1752
Carvone	−19.6648	−26.3592	−24.2672	−25.104	−20.0832
Caryophyllene oxide	−21.7568	−25.9408	−29.7064	−31.38	−22.5936
Cedrelanol	−23.012	−29.7064	−31.7984	−31.38	−25.104
Chamazulene	−22.1752	−35.9824	−30.9616	−32.6352	−26.7776
Chrysanthenone	−21.3384	−23.8488	−26.3592	−25.5224	−18.4096
Cinnamaldehyde	−18.4096	−25.104	−23.012	−25.104	−19.6648
Cinnamic acid	−20.5016	−26.7776	−25.5224	−25.5224	−26.3592
Citral	−17.9912	−23.012	−21.7568	−23.4304	−19.6648
Citronellal	−16.736	−22.1752	−21.3384	−22.5936	−17.1544
Cynaroside	−27.6144	−33.472	−37.2376	−37.656	−28.8696
Decanal	−15.0624	−19.2464	−18.828	−20.5016	−17.1544
Dotriacontanol	−13.3888	−14.644	−19.2464	−18.4096	−15.8992
Elemol	−20.0832	−23.4304	−31.7984	−30.1248	−23.012
Estragole	−19.2464	−25.104	−23.012	−23.8488	−20.5016
Eucalyptol	−19.2464	−23.4304	−22.5936	−24.6856	−17.9912
Farnesol	−20.0832	−25.9408	−25.5224	−23.8488	−21.7568
Farnesyl acetate	−20.5016	−23.4304	−24.2672	−25.5224	−20.5016
Fenchol	−20.0832	−22.5936	−24.2672	−26.3592	−21.7568
Fenchone	−18.828	−21.7568	−24.2672	−24.6856	−18.4096
Geranic acid	−20.0832	−23.8488	−23.012	−23.4304	−19.2464
Geraniol	−18.4096	−24.6856	−22.1752	−21.7568	−18.828
Glutathione	−17.9912	−21.7568	−24.2672	−23.8488	−20.0832
Heneicosane	−17.5728	−18.4096	−20.92	−20.5016	−19.2464
Humulene	−22.5936	−25.104	−28.4512	−31.38	−21.7568
Limonene	−18.828	−27.196	−24.6856	−25.9408	−21.7568
Linalool	−17.1544	−23.012	−22.1752	−23.012	−19.6648
Linalyl acetate	−17.1544	−23.4304	−24.2672	−23.4304	−16.3176
Luteolin	−27.196	−33.0536	−34.3088	−33.8904	−25.104
Myrcene	−17.1544	−24.6856	−20.92	−19.6648	−21.7568
Myrtenal	−19.2464	−23.4304	−23.8488	−25.104	−18.828
Myrtenol	−21.7568	−23.4304	−23.012	−24.6856	−19.2464
Nerol	−18.828	−24.2672	−22.5936	−25.104	−20.5016
Neryl acetate	−20.0832	−23.8488	−25.9408	−24.2672	−20.0832
Neryl propionate	−20.5016	−23.8488	−25.9408	−25.9408	−18.4096
Nonanal	−15.4808	−18.828	−17.9912	−19.6648	−15.8992
Perillaldehyde	−19.6648	−26.3592	−24.6856	−25.9408	−19.6648
Perillene	−18.4096	−25.104	−22.1752	−23.8488	−19.6648
Phellandral	−19.2464	−26.3592	−23.8488	−25.9408	−19.2464
Pinocarvone	−20.0832	−25.104	−25.104	−25.9408	−20.0832
Piperitenone	−19.6648	−25.5224	−25.5224	−25.9408	−21.3384
Piperitone	−20.92	−21.7568	−25.104	−26.7776	−19.6648
Piperonal	−19.2464	−21.7568	−22.5936	−24.2672	−25.5224
Sabinene	−18.828	−25.9408	−23.012	−24.2672	−19.2464
Spathulenol	−26.3592	−27.6144	−29.7064	−34.3088	−23.4304
Terpinolene	−19.6648	−26.7776	−25.5224	−27.196	−22.5936
Thujone	−20.92	−22.1752	−22.5936	−24.2672	−20.0832
Thymol	−20.5016	−27.6144	−23.8488	−27.196	−19.2464
Verbenol	−19.6648	−22.5936	−24.2672	−25.5224	−18.828
Verbenone	−20.0832	−23.012	−24.2672	−26.7776	−18.828
Viridiflorol	−22.5936	−27.196	−30.9616	−31.7984	−22.1752

**Table 3:** Binding affinity of ligands (compounds from cedarwood) interaction with *S. litura* proteins- COX1**,** AGI, ORs, CEHs, and CSPs.

Name of the compound	Binding affinity in Kcal/mol				
	CSPs	AGI	ORs	CEHs	COX1
Hinokiflavone	−35.1456	−38.4928	−40.5848	−44.7688	−35.1456
Cupressuflavone	−29.288	−38.0744	−40.1664	−35.9824	−28.8696
Robustaflavone	−37.2376	−42.6768	−39.748	−41.0032	−34.3088
Agathisflavone	−33.472	−39.3296	−39.748	−36.8192	−33.472
Amentoflavone	−33.472	−39.748	−45.1872	−44.7688	−31.38
Carvacrol	−20.5016	−25.5224	−25.104	−26.3592	−22.1752
Thymol	−20.5016	−27.6144	−23.8488	−27.196	−19.2464
Cedrol	−23.8488	−26.7776	−29.288	−32.6352	−23.8488
Podofilox	−26.3592	−30.9616	−28.8696	−30.5432	−25.104

**Table 4:** Consolidated best binding affinity ligands (compounds from lemon grass and cedarwood) with *S. litura* proteins—COX1**,** AGI, ORs, CEHs, and CSPs.

Affinity of best ligand compounds with each protein					
Name of the protein	Lemongrass		Cedarwood		Chloropyriphos
	Compounds	Binding affinity (KJ/mol)	Compounds	Binding affinity (KJ/mol)	Binding affinity (KJ/mol)
CSPs	Spathulenol	−27.6144	Robustaflavone	−37.2376	−19.2464
AGI	Chamazulene	−35.9824	Robustaflavone	−42.6768	−20.92
ORs	Cynaroside	−37.2376	Amentoflavone	−45.1872	−23.4304
COX1	Cynaroside	−37.656	Hinokiflavone	−44.7688	−21.7568
CEHs	Cynaroside	−28.8696	Hinokiflavone	−35.1456	−20.0832

**Table 5:** Docking interaction profile residues of *S. litura* proteins—COX1**,** AGI, ORs, CEHs, and CSPs with consolidated best binding affinity ligand compounds from lemon grass and cedarwood; and also, with reference molecule chlorpyrifos.

Docking interaction profile						
Name of the protein	Hydrogen bond interactions			Hydrophobic interactions		
COX1	Lemongrass	Cedarwood	Chlorpyrifos	Lemongrass	Cedarwood	Chlorpyrifos
	GLU 234, ALA 179, THY 183, VAL 235, PRO 233	GLU 234	THR 183	ILE 182, ILE 67, MET 66, PHE 176, LEU 175, LEU 186	THR 183, ILE 67, MET 66, VAL 62, MET 63, ILE 58, MET 57, VAL 235, PRO 233, TRP 178, ILE 182	MET 66, ALA 179, ILE 182
AGI	Lemongrass	Cedarwood	Chlorpyrifos	Lemongrass	Cedarwood	Chlorpyrifos
	–	ARG 366, HIS 362, GLN 335, SER 363, GLU 406, ARG 334	–	ALA204, PRO 193, ILE 173, GLU 172, ALA 171, PHE 211, VAL 190, VAL 192	THR 371, HIS 367, ARG 409, GLY 407, ASN 277, TYR 361, SER 368	ALA 204, PRO 193, TRP 320, PHE 211, SER 207, ALA 171
CEHs	Lemongrass	Cedarwood	Chlorpyrifos	Lemongrass	Cedarwood	Chlorpyrifos
	TYR 139, HIS 443, TYR 397, SER 333	TYR 139, ASN 351, GLN 354, TYR 397, SER 207, PHE 133	–	GLY 126, LEU138, ILE 131, TRP 239, TYR 396, ALA 330, SER 207, SER 206, GLY 127, LEU 447	GLU 90, GLY 126, GLY 127, HIS 443, ILE 128, TRP 239, ARG 290, LEU 138, ILE 131, GLY 132	LYS 223, GLY 317, SER 315, SER 220, HIS 221, LEU 316
ORs	Lemongrass	Cedarwood	Chlorpyrifos	Lemongrass	Cedarwood	Chlorpyrifos
		THR 444	ASN 88, ARG 150		LEU 206, PRO 428, TYR 424, CYS 29, CYS 433, ILE 427, CYS 434, VAL 213, ARG 205, ILE 440, TYR 112, PHE 420	ASN 462, ARG 398, ILE 85, ILE 91, ALA 402
CSPs	Lemongrass	Cedarwood	Chlorpyrifos	Lemongrass	Cedarwood	Chlorpyrifos
	TYR 9, LYS 8	LYS 36, TYR 27	GLN 63	ASP 10, GLY 66, ALA 62, HIS 47, GLN 63, GLU 43	SER 38, ALA 26, GLU 16, LEU 23, GLU 3, ASP 13, TYR 5, LYS 8, GLU 40	THR 60, TYR 9, LYS 8, GLU 43, ALA 62, ASP 10

### Grouped ligand-protein docking

**Fig. 5:**
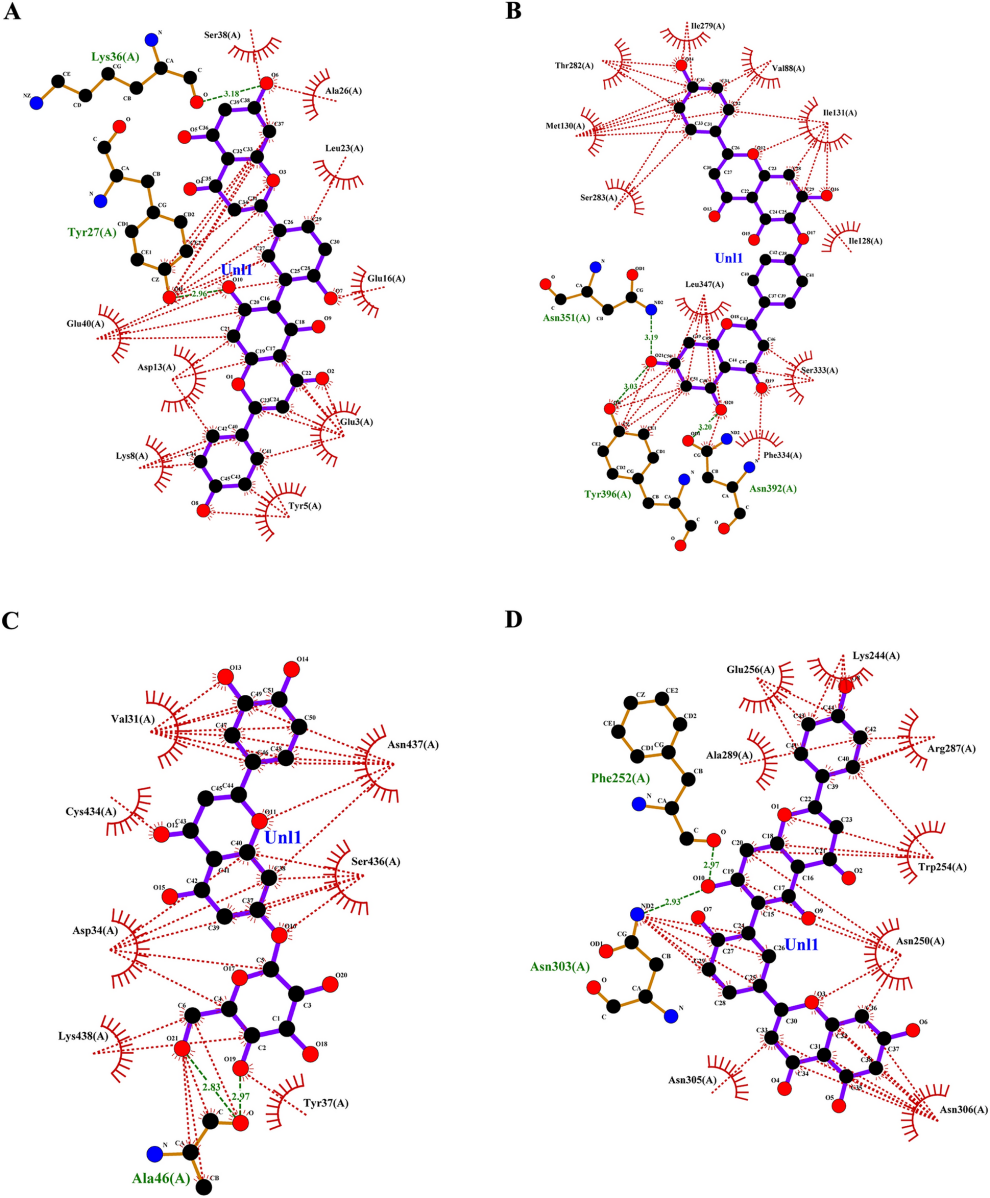

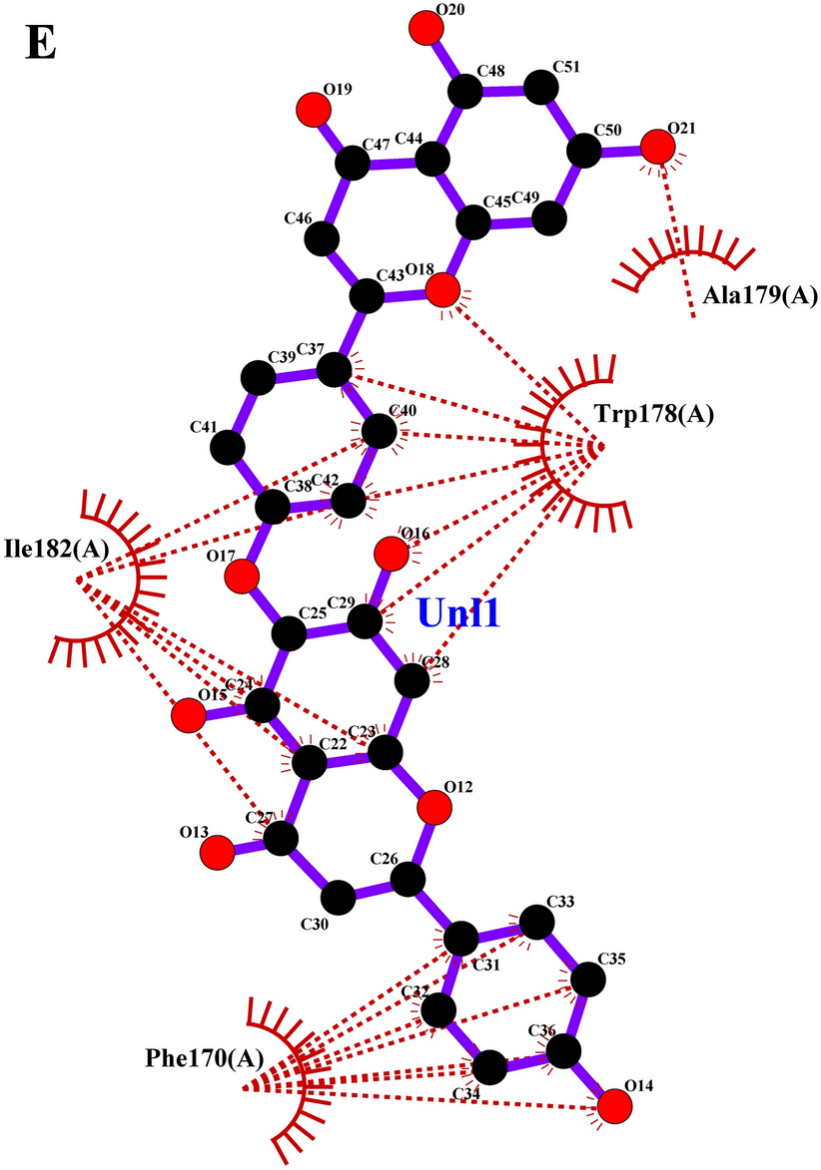
(**A**–**E**) 2D interaction profile of combined ligand compounds from lemongrass and cedarwood with *S. litura* proteins—COX1, AGI, ORs, CEHs and CSPs using ligplot plus. (**A**) The 2D interaction of compounds spathulenol + robustaflavone from lemongrass and cedarwood with chemosensory protein of *S. litura*. (**B**) The 2D interaction of compounds cynaroside + hinokiflavone from lemongrass and cedarwood with CEHs of *S. litura*. (**C**) The 2D interaction of compounds cynaroside + amentoflavone from lemongrass and cedarwood with octopamine receptor protein of *S. litura*. (**D**) The 2D interaction of compounds chamazulene + robustaflavone from lemongrass and cedarwood with AGI of *S. litura*. (**E**) The 2D interaction of compounds cynaroside + hinokiflavone from lemongrass and cedarwood with cytochrome c oxidase of *S. litura*.

**Fig. 6:**
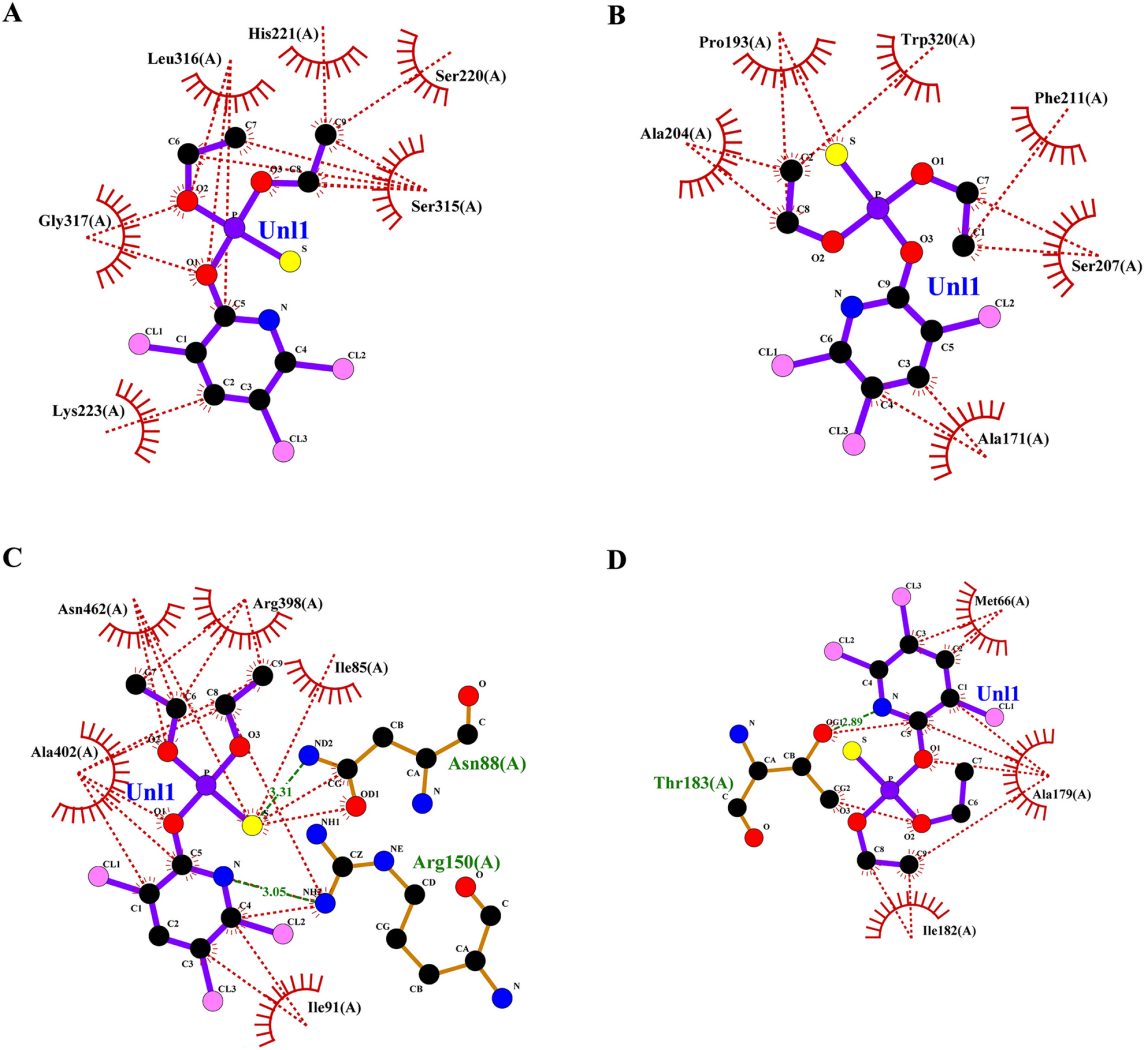

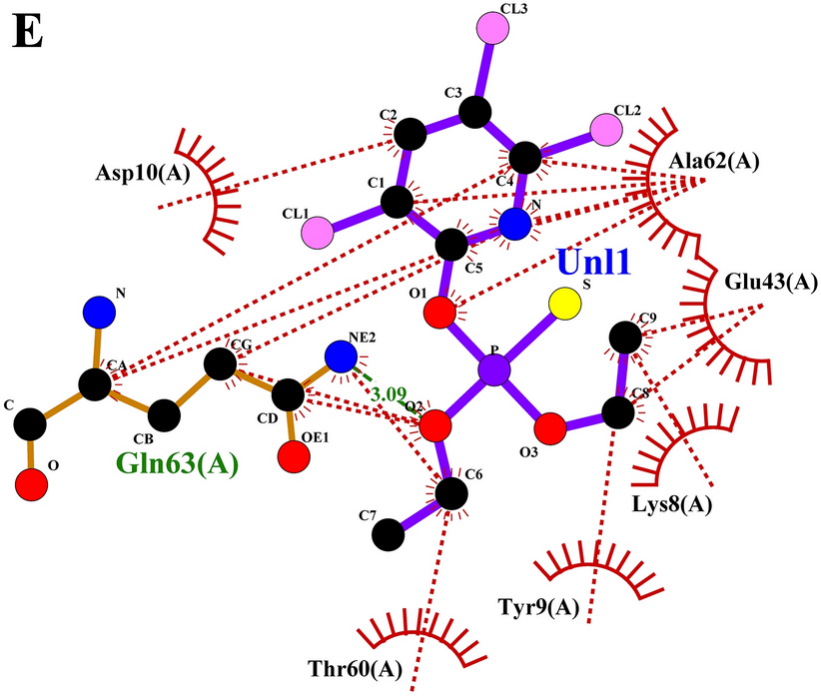
(**A**–**E**) The 2D interaction profile of chlorpyrifos with *S. litura* proteins—COX1, AGI, ORs, CEHs, and CSPs from the ligplot plus. (**A**) The 2D interaction of compounds chlorpyrifos with cytochrome c oxidase of *S. litura*. (**B**) The 2D interaction of compounds chlorpyrifos with AGI of *S. litura*. (**C**) The 2D interaction of compounds chlorpyrifos with octopamine recrptor of *S. litura*. (**D**) The 2D interaction of compounds chlorpyrifos with CEHs and of *S. litura*. (**E**) The 2D interaction of compounds chlorpyrifos with cytochrome c oxidase of *S. litura*.

Later the grouped docking analysis of the best binding affinity ligands from lemongrass and cedarwood with key *S. litura* proteins reveals significant interactions that may enhance the understanding of bioinsecticide development. The combined binding affinities indicate promising synergistic effects of the ligands. For the CSPs, the combination of spathulenol and robustaflavone yielded a binding affinity of −51.83976 kJ/mol, suggesting a strong interaction that could disrupt olfactory signaling in the pest. In the case of AGI, chamazulene paired with robustaflavone demonstrated a binding affinity of −59.24544 kJ/mol, indicating effective inhibition that may hinder carbohydrate metabolism. The ORs, crucial for neuromodulation in insects, showed a binding affinity of −56.6932 kJ/mol with the combination of cynaroside and amentoflavone, potentially affecting the pest’s behavior. For CEHs, the combination of cynaroside and hinokiflavone achieved a binding affinity of −61.0864 kJ/mol, indicating a strong potential for enzyme inhibition. Lastly, the COX1 complex exhibited a binding affinity of −46.98632 kJ/mol with cynaroside and hinokiflavone, which may impair energy production in the pest. These findings highlight the potential of combined essential oil formulations to target multiple proteins in *S. litura*, offering a promising avenue for the development of effective bioinsecticides. The 2D interaction profile of combined ligand compounds from lemongrass and cedarwood with *S. litura* proteins—COX1**,** AGI, ORs, CEHs, and CSPs from the ligplot plus were represented in (Fig. 5A–E). Table 6, grouped ligand docking binding affinity of combined compounds from lemongrass and cedarwood with a comparison of chlorpyrifos against *S. litura* proteins- COX1**,** AGI, ORs, CEHs, and CSPs were represented. In comparing the binding affinities of chlorpyrifos with lemongrass and cedarwood compounds, the natural combinations exhibit significantly stronger interactions with various proteins, indicating their potential as effective alternatives. For the CSPs, spathulenol and robustaflavone show a binding affinity of −51.83976 kJ/mol, compared to chlorpyrifos at −19.2464 kJ/mol. Similarly, chamazulene and robustaflavone bind more effectively to AGI (−59.24544 vs. −20.92 kJ/mol), and cynaroside and amentoflavone show stronger binding to ORs s (−56.6932 vs. −23.4304 kJ/mol). For COX1 and CEHs, natural compounds exhibit higher affinities (−61.0864 and −46.98632 kJ/mol, respectively) compared to chlorpyrifos (−21.7568 and −20.0832 kJ/mol). These results suggest that lemongrass and cedarwood compounds may offer more potent pest control while being safer alternatives to chlorpyrifos. The 2D interaction profile of chlorpyrifos with *S. litura* proteins—COX1**,** AGI, ORs, CEHs, and CSPs from the ligplot plus were represented in (Fig. 6A–E). Grouped docking interaction profile of *S. litura* proteins with consolidated best-binding affinity ligand compounds from lemongrass and cedarwood, the reference molecule chlorpyrifos were represented in (Table 7). From the Table 7, natural compound combinations (cynaroside + hinokiflavone, chamazulene + robustaflavone, spathulenol + robustaflavone, and cynaroside + amentoflavone) demonstrate a more favourable interaction profile than chlorpyrifos when docked with the key *S. litura* proteins: COX1, AGI, CEHs, ORs, and CSPs. These natural combinations form stronger and more numerous interactions with critical residues, enhancing their potential to bind and disrupt the function of these proteins. For example, cynaroside + hinokiflavone forms strong hydrophobic interactions with residues like PHE 170, ILE 182, TRP 178, and ALA 179 in COX1, and with LEU 347, PHE 334, ILE 128, and ILE 131 in CEHs, suggesting a more stable and effective binding compared to chlorpyrifos, which forms fewer interactions with residues like MET 66, ALA 179, and ILE 182. Similarly, chamazulene + robustaflavone establishes significant hydrogen bonds with residues such as PHE 252 and ASN 303 in AGI, while chlorpyrifos shows no hydrogen bonding with this enzyme, indicating a weaker potential for inhibiting the protein. The natural compound pair also interacts hydrophobically with residues like ALA 289, GLU 256, and TRP 254, further enhancing its binding stability. In the ORs, cynaroside + amentoflavone forms a critical hydrogen bond with ALA 46 and engages in hydrophobic interactions with residues such as TYR 37, LYS 438, and ASP 34, which could disrupt receptor signaling more effectively than chlorpyrifos, which binds weakly to residues like ASN 88 and ARG 150. Lastly, spathulenol + robustaflavone demonstrates strong hydrogen bonds with LYS 36 and TYR 27 in the CSPs, and forms hydrophobic contacts with key residues such as SER 38, LEU 23, and GLU 16, whereas chlorpyrifos shows limited interaction with residues like GLN 63. Overall, the natural compounds exhibit more extensive and stronger interactions with key residues across all proteins analyzed.

**Table 6:** Grouped ligand docking binding affinity of combined compounds from lemon grass and cedarwood with comparison chlorpyrifos against *S. litura* proteins- COX1**,** AGI, ORs, CEHs, and CSPs.

Group docking of best binding affinity ligands with each protein			Chlorpyrifos binding affinity (KJ/mol)
Name of the proteins	Lemongrass + Cedarwood		
	Compounds	Binding affinity (KJ/mol)	
CSPs	Spathulenol + Robustaflavone	−51.83976	−19.2464
AGI	Chamazulene + Robustaflavone	−59.24544	−20.92
ORs	Cynaroside + Amentoflavone	−56.6932	−23.4304
CEHs	Cynaroside + Hinokiflavone	−61.0864	−21.7568
COX1	Cynaroside + Hinokiflavone	−46.98632	−20.0832

**Table 7:** Grouped docking interaction profile residues of *S. litura* proteins—COX1**,** AGI, ORs, CEHs, and CSPs with consolidated best binding affinity ligand compounds from lemon grass and cedarwood; and also, with reference molecule chlorpyrifos.

Grouped docking interaction profile				
Name of the protein	Hydrogen bond interactions		Hydrophobic interactions	
COX1	Cynaroside + Hinokiflavone	Chlorpyrifos	Cynaroside + Hinokiflavone	Chlorpyrifos
	–	THR 183	PHE 170, ILE 182, TRP 178, ALA 179	MET 66, ALA 179, ILE 182
AGI	Chamazulene + Robustaflavone	Chlorpyrifos	Chamazulene + Robustaflavone	Chlorpyrifos
	PHE 252, ASN 303	–	ALA 289, GLU 256, LYS 244, ARG 287, TRP 254, ASN 250, ASN 306, ASN 305	ALA 204, PRO 193, TRP 320, PHE 211, SER 207, ALA 171
CEHs	Cynaroside + Hinokiflavone	Chlorpyrifos	Cynaroside + Hinokiflavone	Chlorpyrifos
	ASN 351, TYR 396 ASN 392	–	LEU 347, PHE 334, SER 333, ILE 128, ILE 131, SER 283, MET 130, VAL 188, ILE 279, THR 282	LYS 223, GLY 317, SER 315, SER 220, HIS 221, LEU 316
ORs	Cynaroside + Amentoflavone	Chlorpyrifos	Cynaroside + Amentoflavone	Chlorpyrifos
	ALA 46	ASN 88, ARG 150	TYR 37, LYS 438, ASP 34, SER 436, CYS 434, VAL 31, ASN 437	ASN 462, ARG 398, ILE 85, ILE 91, ALA 402
CSPs	Spathulenol + Robustaflavone	Chlorpyrifos	Spathulenol + Robustaflavone	Chlorpyrifos
	LYS 36, TYR 27	GLN 63	SER 38, ALA 26, LEU 23, GLU 16, GLU 40, ASP 13, GLU 3, LYS 8, TYR 5	THR 60, TYR 9, LYS 8, GLU 43, ALA 62, ASP 10

The comparative docking analysis of natural compounds from lemongrass and cedarwood against key Spodoptera litura proteins revealed significantly higher binding affinities than the conventional insecticide chlorpyrifos, indicating their potential as effective bioinsecticides. These findings align with prior studies that explored the inhibitory effects of essential oil constituents on critical insect biochemical pathways. Awad et al.^48^ demonstrated that terpinen-4-ol, (Z)-citral, and α-pinene, the primary components of marjoram, citronella, and rosemary essential oils, exhibited strong binding affinities with glutathione S-transferase (GST), suggesting their potential to interfere with detoxification mechanisms in insects. Similarly, Aathmanathan et al.^49^ identified spinosyn A and milbemycin A4 as potent inhibitors of the vitellogenin receptor (VgR), a key protein in reproductive processes, highlighting their role in disrupting pest fertility. The present study extends these findings by showing that compounds such as spathulenol, robustaflavone, chamazulene, and cynaroside exhibited stronger and more stable interactions with CSPs, AGI, and ORs than chlorpyrifos. Moreover, the observed hydrogen bonding and hydrophobic interactions with critical residues reinforce the structural stability of these ligand-protein complexes, further supporting the insecticidal efficacy of natural compounds. Collectively, these studies underscore the potential of plant-derived bioactive molecules as eco-friendly alternatives to synthetic pesticides, offering targeted mechanisms of pest control while mitigating environmental toxicity.

### Simulation studies

**Fig. 7:**
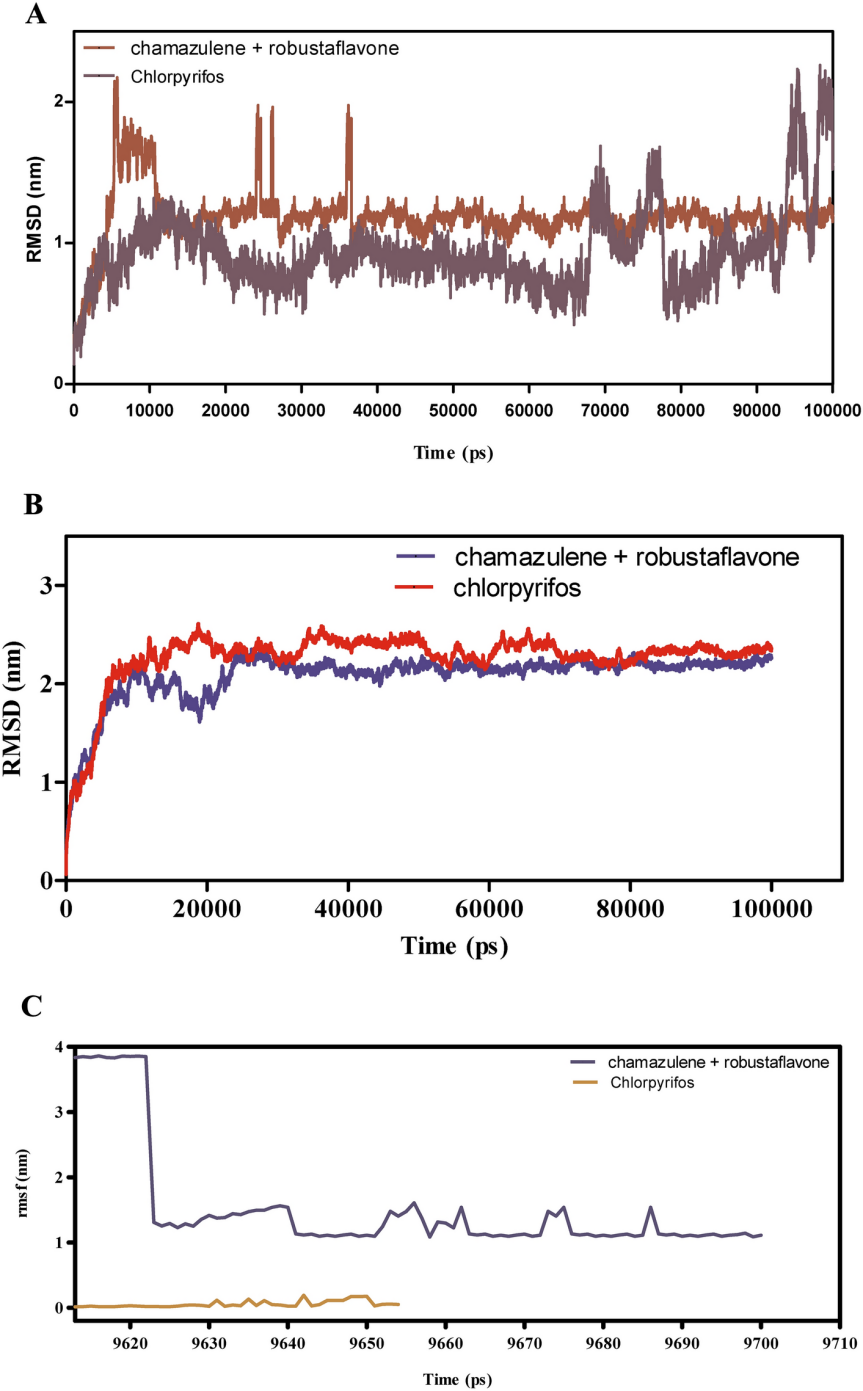

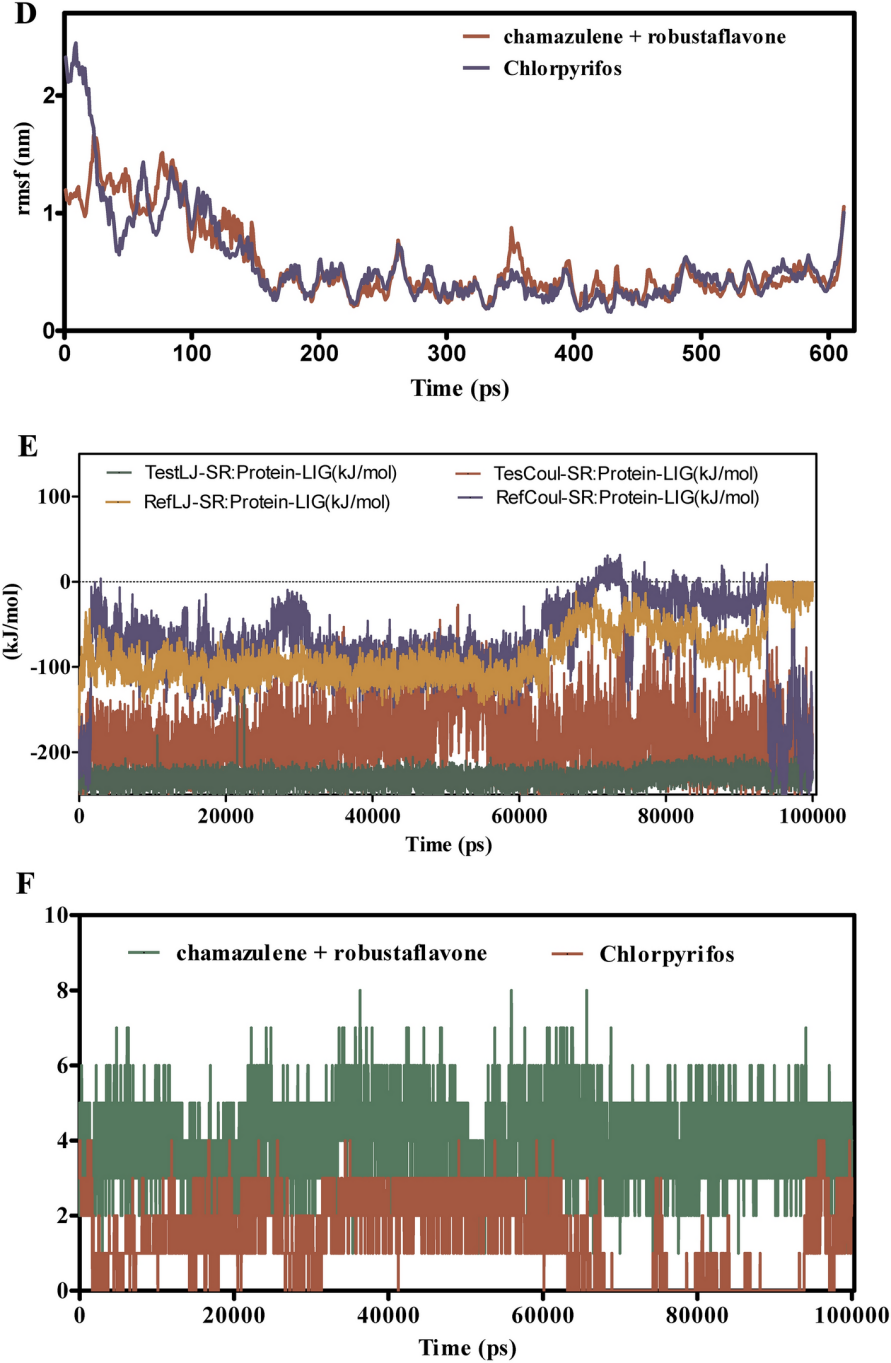
(**A**–**F**) The molecular dynamic simulations of AGI with ligands- chamazulene + robustoflavone and chlorpyrifos (reference) for 100 ns. The Root mean square deviation (RMSD), Root mean square fluctuation (RMSF), interaction energies, and Hydrogen bond interactions of ligands- chamazulene + robustoflavone and chlorpyrifos (reference) for 100 ns were represented. (**A**) Root mean square deviation (RMSD) of ligands- chamazulene + robustoflavone and chlorpyrifos (reference) for 100 ns. (**B**) Root mean square deviation (RMSD) of AGI interaction with chamazulene + robustoflavone and chlorpyrifos (reference) for 100 ns. (**C**) Root mean square fluctuation (RMSF) of ligands chamazulene + robustoflavone and chlorpyrifos (reference) interaction with receptor for 100 ns. (**D**) Root mean square fluctuation (RMSF) of AGI interaction with chamazulene + robustoflavone and chlorpyrifos (reference) for 100 ns. (**E**) Lennard–Jones interactions and Coulomb interactions energies of AGI receptor and ligands- chamazulene + robustoflavone and chlorpyrifos (reference) for 100 ns. (**F**) Hydrogen bond interactions of chamazulene + robustoflavone and chlorpyrifos (reference) with AGI receptor for 100 ns.

The molecular dynamics (MD) simulations presented in (Fig. 7A–F) evaluate the binding stability, flexibility, and interaction energies of chamazulene + robustoflavone versus chlorpyrifos with alpha-glucosidase over a 100 ns trajectory. The root mean square deviation (RMSD) analysis in Fig. 7A reveals that chamazulene + robustoflavone maintains an average RMSD of approximately 1.5 Å, indicating a stable binding pose with minimal positional shifts. In contrast, chlorpyrifos exhibits higher RMSD fluctuations around 2.0 Å, suggesting a less stable interaction within the binding pocket. Correspondingly, Fig. 7B shows that the RMSD of AGI itself remains steady at approximately 1.2 Å when bound to chamazulene + robustoflavone, compared to an average of 1.6 Å with chlorpyrifos, which occasionally spikes. This indicates that chlorpyrifos induces more conformational perturbations in the enzyme, potentially compromising binding stability. Figure 7C demonstrates that chamazulene + robustoflavone exhibits a low Root mean square fluctuation (RMSF) of around 1.5 Å or less, reflecting minimal atomic movement and strong retention within the binding site. Chlorpyrifos, however, shows higher RMSF values up to 2.5 Å, indicating greater flexibility and weaker interactions. Additionally, Fig. 7D highlights that AGI residues interacting with chamazulene + robustoflavone display lower RMSF (~ 1.0 Å) compared to those with chlorpyrifos (~ 2.0 Å), further supporting the notion of a more stabilized enzyme structure upon binding with chamazulene + robustoflavone. The interaction energy analysis in Fig. 7E reveals that chamazulene + robustoflavone forms more favorable Lennard–Jones and coulomb interactions, with coulomb energies averaging around −150 kJ/mol, compared to chlorpyrifos’s -100 kJ/mol. This signifies stronger electrostatic attractions facilitating effective binding. Figure 7F shows that chamazulene + robustoflavone consistently forms 3–5 hydrogen bonds with bond lengths around 2.0 Å, whereas chlorpyrifos forms fewer (1–2) and longer, more variable hydrogen bonds (2.5–3.0 Å). The robust hydrogen bonding network of chamazulene + robustoflavone contributes significantly to its superior binding stability and affinity. Collectively, chamazulene + robustoflavone demonstrate enhanced binding stability, reduced flexibility, stronger interaction energies, and more stable hydrogen bonding with AGI compared to chlorpyrifos. These characteristics underscore the potential of chamazulene + robustoflavone as a more effective inhibitor of AGI.

**Fig. 8:**
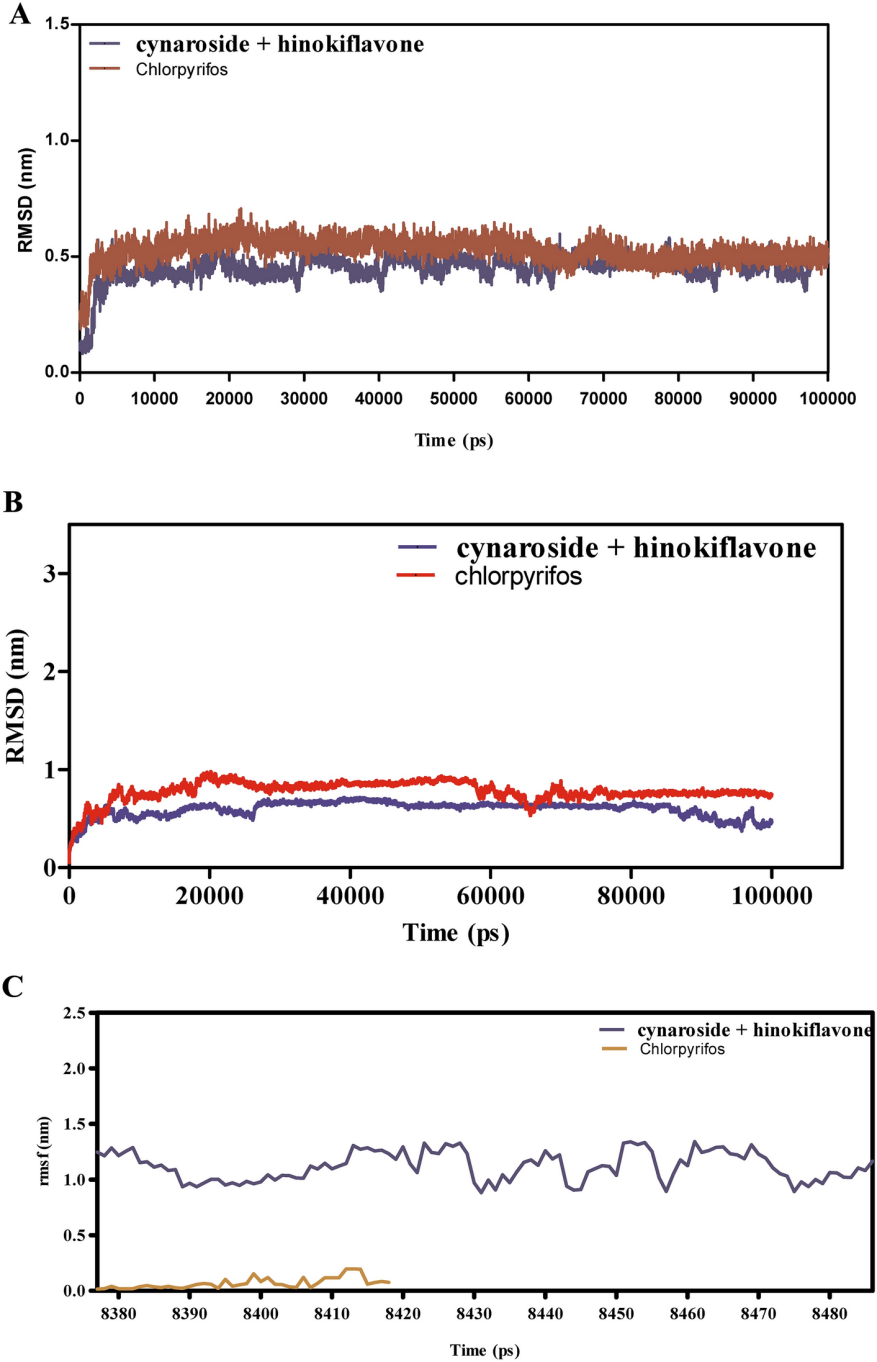

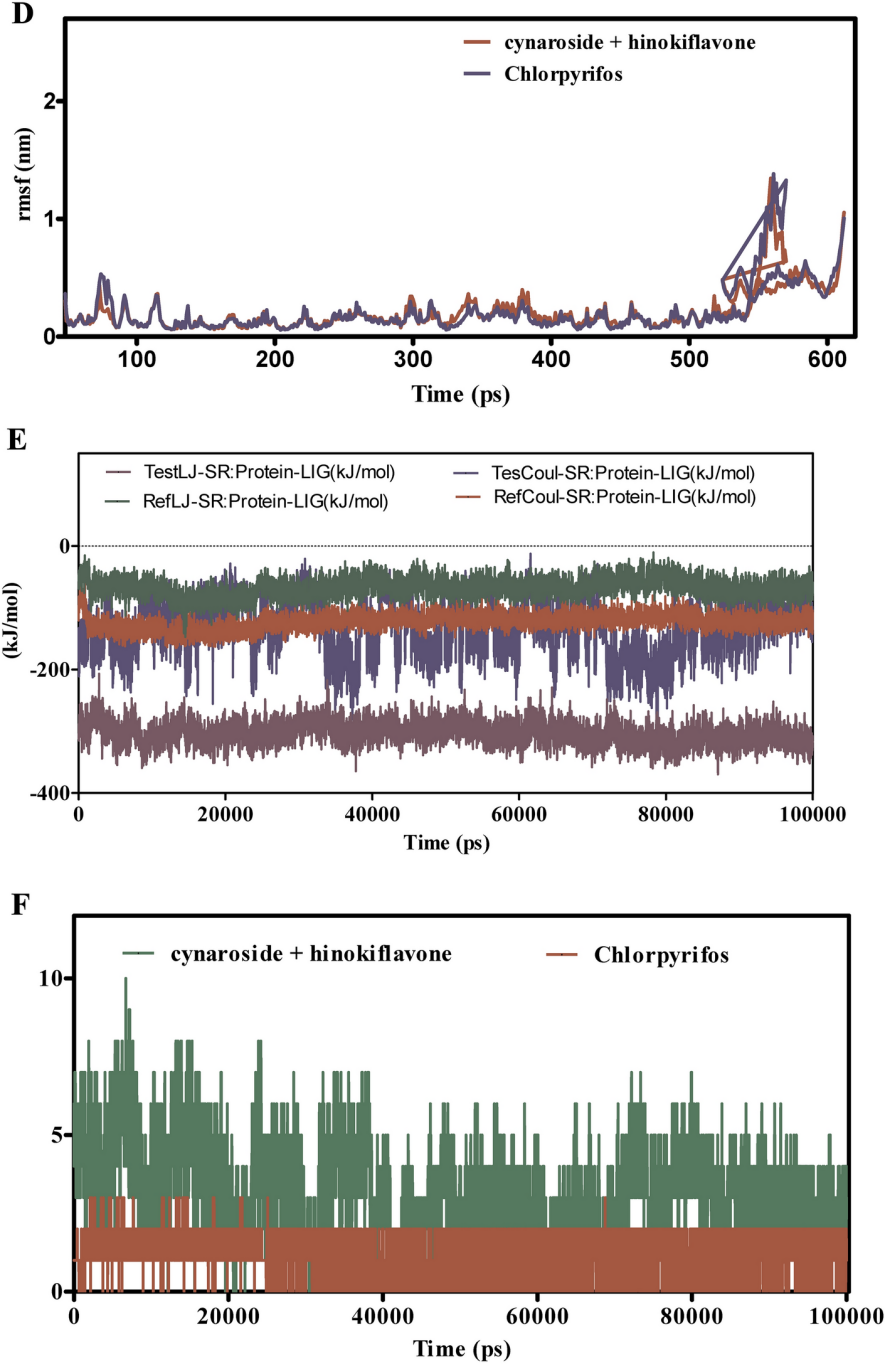
(**A**–**F**) The molecular dynamic simulations of CEHs with ligands- cynaroside + hinkoflavone and chlorpyrifos (reference) for 100 ns. The Root mean square deviation (RMSD), Root mean square fluctuation (RMSF), interaction energies, and Hydrogen bond interactions of ligands- cynaroside + hinkoflavone and chlorpyrifos (reference) for 100 ns were represented. (**A**) Root mean square deviation (RMSD) of ligands- cynaroside + hinkoflavone and chlorpyrifos (reference) for 100 ns. (**B**) Root mean square deviation (RMSD) of CEHs interaction with cynaroside + hinkoflavone and chlorpyrifos (reference) for 100 ns. (**C**) Root mean square fluctuation (RMSF) of ligands cynaroside + hinkoflavone and chlorpyrifos (reference) interaction with receptor for 100 ns. (**D**) Root mean square fluctuation (RMSF) of CEHs interaction with cynaroside + hinkoflavone and chlorpyrifos (reference) for 100 ns. (**E**) Lennard–Jones interactions and Coulomb interactions energies of CEHs receptor and ligands- cynaroside + hinkoflavone and chlorpyrifos (reference) for 100 ns. (**F**) Hydrogen bond interactions of cynaroside + hinkoflavone and chlorpyrifos (reference) with CEHs receptor for 100 ns.

Figure 8A–F explores the binding dynamics of cynaroside + hinkoflavone versus chlorpyrifos with CEHs over a 100 ns simulation. RMSD analyses in Fig. 8A,B indicate that cynaroside + hinkoflavone maintains stable RMSD values between 1.5–2.0 Å, suggesting tight binding and minimal structural deviation. In contrast, chlorpyrifos shows greater RMSD variability, indicative of weaker and less stable binding. Additionally, when bound to COX1, cynaroside + hinkoflavone results in a controlled RMSD peaking around 2.2 Å, whereas chlorpyrifos induces wider fluctuations, implying a less stable interaction. Figure 8C reveals that cynaroside + hinkoflavone exhibits lower RMSF values, typically around 1.0 Å, indicating reduced atomic flexibility and stronger anchoring within the binding site. Conversely, chlorpyrifos displays RMSF values exceeding 1.5 Å, suggesting higher residue mobility and less rigid binding. Figure 8D further supports this by showing that residues interacting with cynaroside + hinkoflavone have lower fluctuations, maintaining the protein’s structural integrity. The interaction energy profile in Fig. 8E shows that cynaroside + hinkoflavone forms more favorable and stable Lennard–Jones and Coulomb interactions compared to chlorpyrifos, which exhibits erratic energy readings indicative of inconsistent binding. Figure 8F illustrates that cynaroside + hinkoflavone consistently forms more hydrogen bonds throughout the simulation, enhancing the stability of the ligand–protein complex. In contrast, chlorpyrifos forms fewer and less stable hydrogen bonds, correlating with its weaker binding profile. Cynaroside + hinkoflavone outperforms chlorpyrifos in terms of binding stability, reduced flexibility, favorable interaction energies, and robust hydrogen bonding with CEHs. These findings position cynaroside + hinkoflavone as a more effective ligand for inhibiting CEHs.

**Fig. 9:**
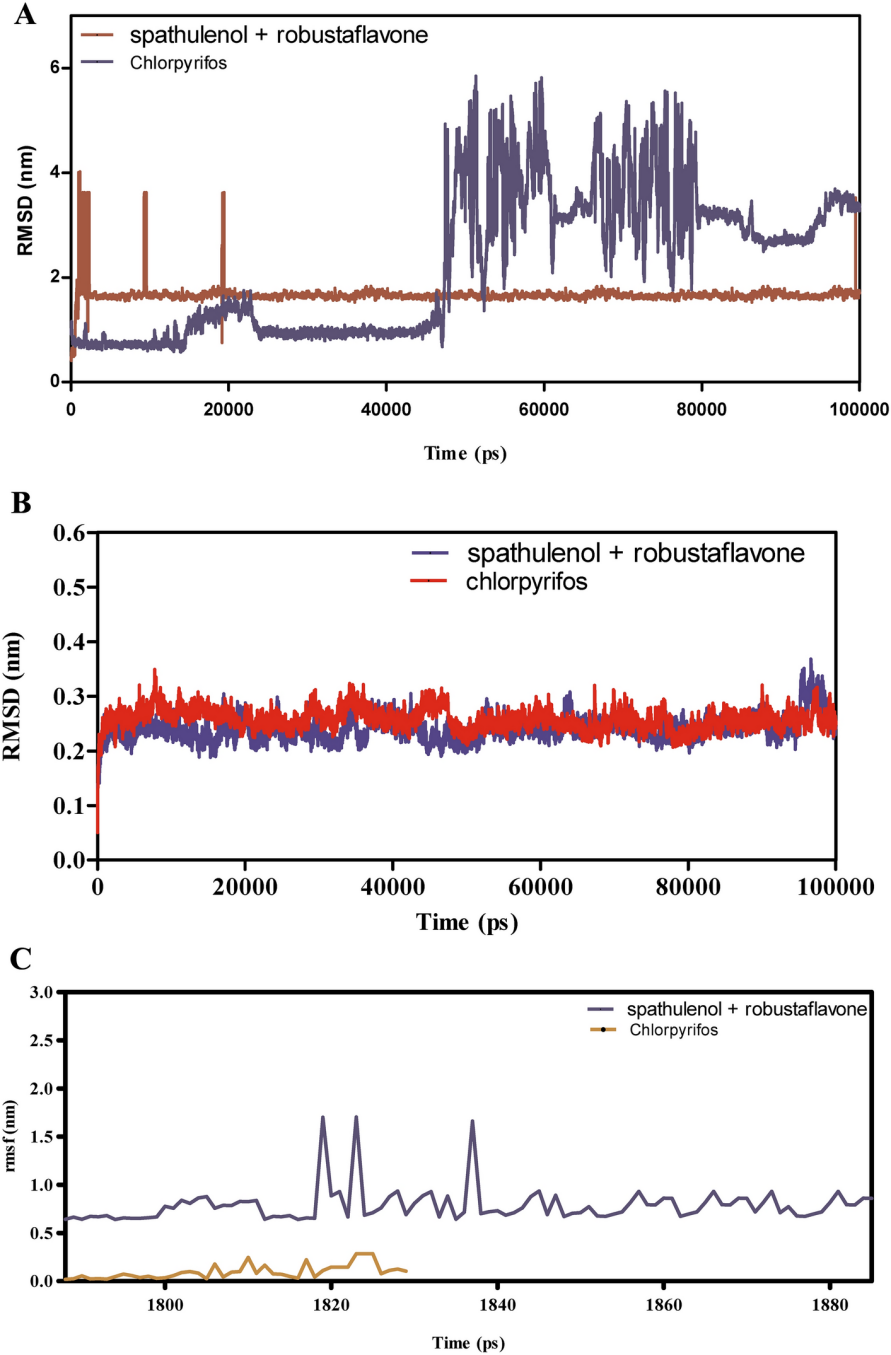

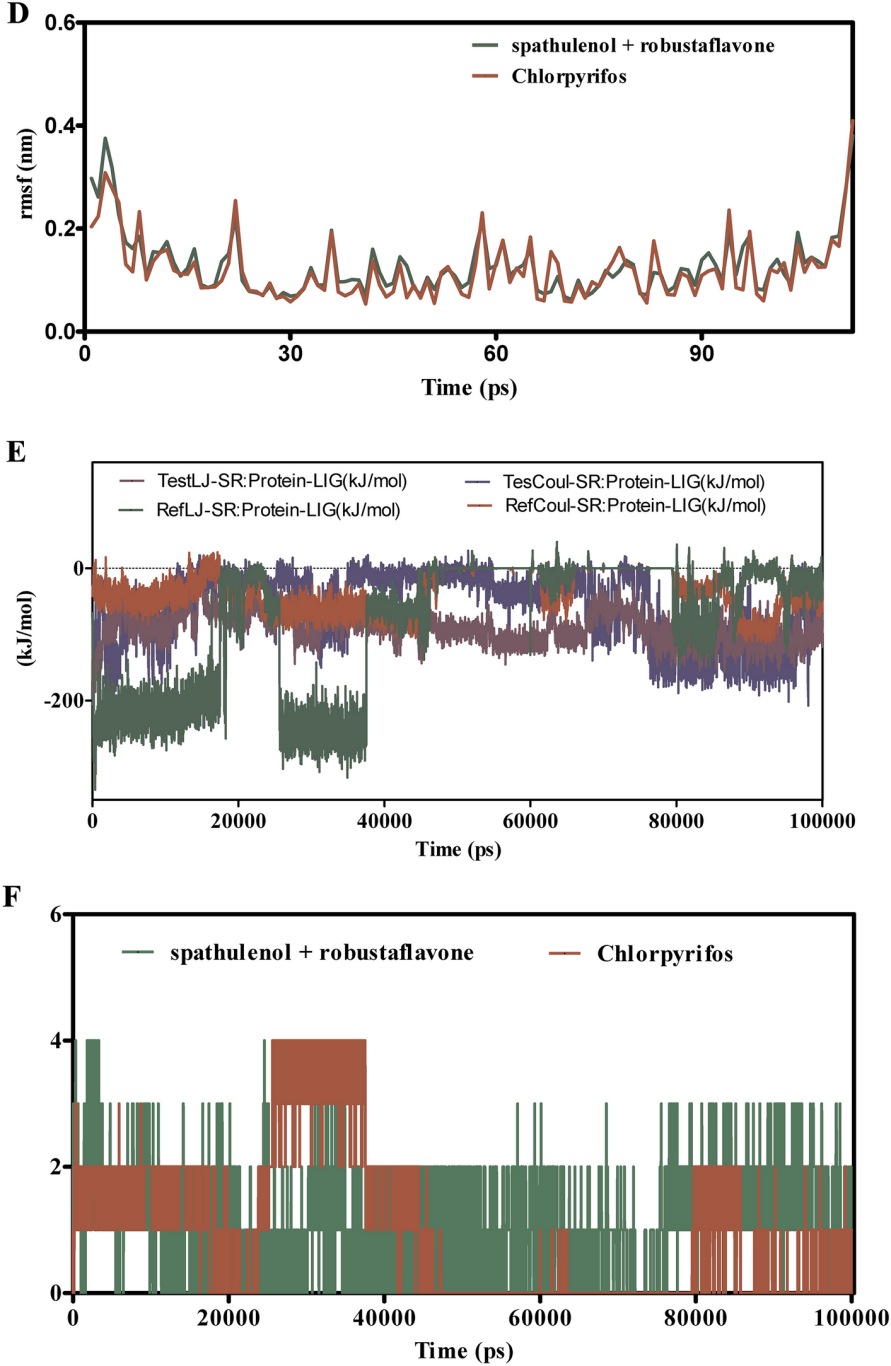
(**A**–**F**) The molecular dynamic simulations of CSPs with ligands- spathulenol + robustaflavone and chlorpyrifos (reference) for 100 ns. The Root mean square deviation (RMSD), Root mean square fluctuation (RMSF), interaction energies, and Hydrogen bond interactions of ligands- spathulenol + robustaflavone and chlorpyrifos (reference) for 100 ns were represented. (**A**) Root mean square deviation (RMSD) of ligands- spathulenol + robustaflavone and chlorpyrifos (reference) for 100 ns. (**B**) Root mean square deviation (RMSD) of CSPs interaction with spathulenol + robustaflavone and chlorpyrifos (reference) for 100 ns. (**C**) Root mean square fluctuation (RMSF) of ligands spathulenol + robustaflavone and chlorpyrifos (reference) interaction with receptor for 100 ns. (**D**) Root mean square fluctuation (RMSF) of CSPs interaction with spathulenol + robustaflavone and chlorpyrifos (reference) for 100 ns. (**E**) Lennard–Jones interactions and Coulomb interactions energies of CSPs receptor and ligands- spathulenol + robustaflavone and chlorpyrifos (reference) for 100 ns. (**F**) Hydrogen bond interactions of spathulenol + robustaflavone and chlorpyrifos (reference) with CSPs receptor for 100 ns.

Figure 9A–F presents the MD simulation results comparing spathulenol + robustaflavone with chlorpyrifos in their interactions with CSPs over 100 ns. The RMSD analysis in Fig. 9A,B indicates that the spathulenol + robustaflavone complex maintains RMSD values between 1.5 to 2.5 Å, reflecting a stable binding mode. In contrast, chlorpyrifos complexes exhibit higher RMSD values ranging from 2.0 to 3.5 Å, suggesting greater structural variations and less stable binding. Figure 9C shows that the RMSF values for key binding site residues in the spathulenol + robustaflavone complex remain between 1.0 to 1.8 Å, indicating limited flexibility and a more rigid binding conformation. Conversely, chlorpyrifos-bound complexes display RMSF values between 1.5 to 2.3 Å, indicating increased residue flexibility and potentially weaker interactions. Interaction energies depicted in Fig. 9E demonstrate that spathulenol + robustaflavone forms stronger Lennard–Jones interactions (−15 to −25 kcal/mol) and more favorable Coulomb energies (−10 to −15 kcal/mol) compared to chlorpyrifos (−10 to −20 kcal/mol for Lennard–Jones and −8 to −12 kcal/mol for Coulomb). Figure 9F illustrates that spathulenol + robustaflavone consistently forms more stable hydrogen bonds with bond distances between 2.8 to 3.2 Å, whereas chlorpyrifos forms fewer and more transient hydrogen bonds with longer distances (3.0 to 3.5 Å). Spathulenol + robustaflavone exhibits superior binding stability, reduced flexibility, stronger interaction energies, and more stable hydrogen bonding with CSPs compared to chlorpyrifos. These attributes suggest that spathulenol + robustaflavone forms a more effective and stable ligand-protein complex.

**Fig. 10:**
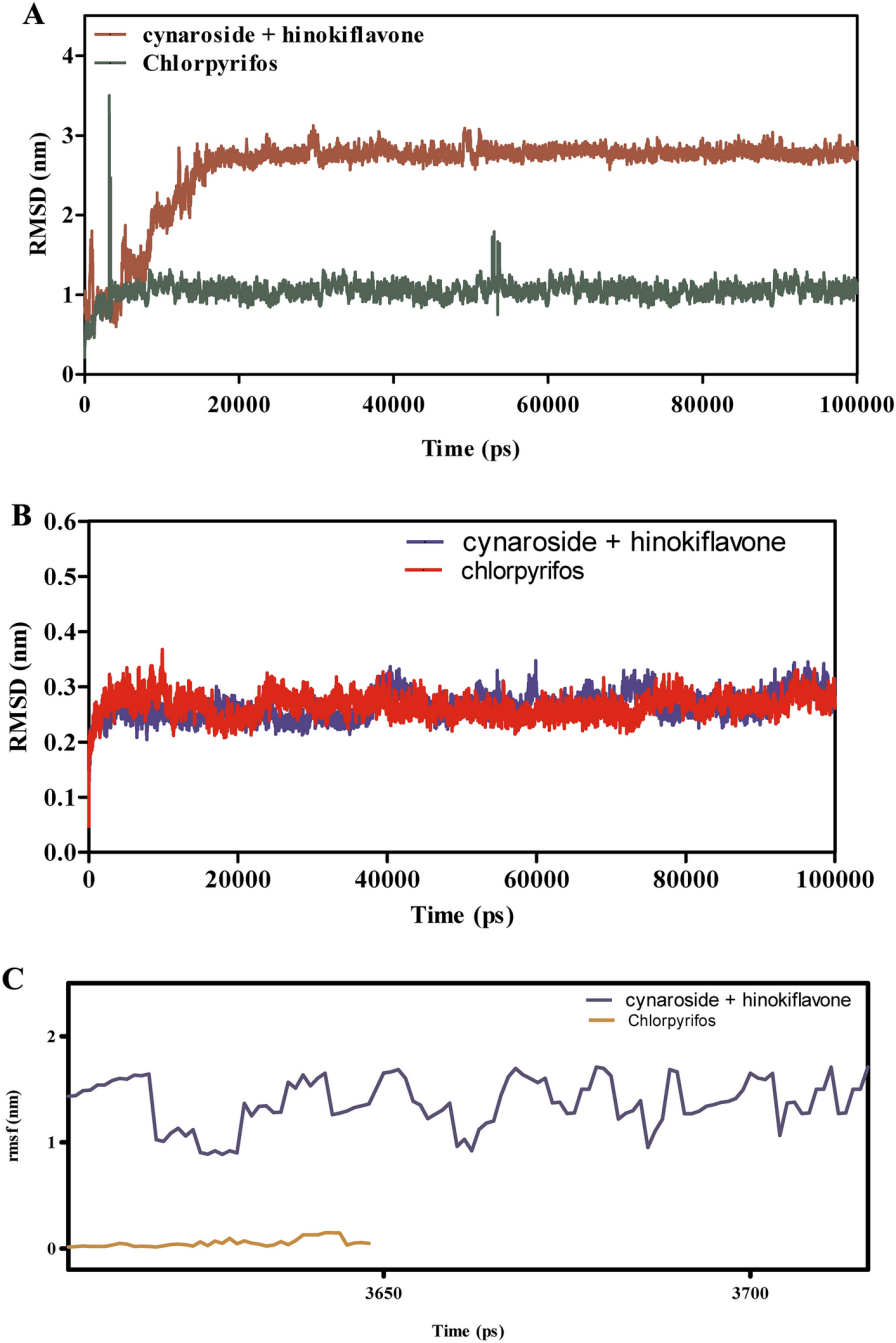

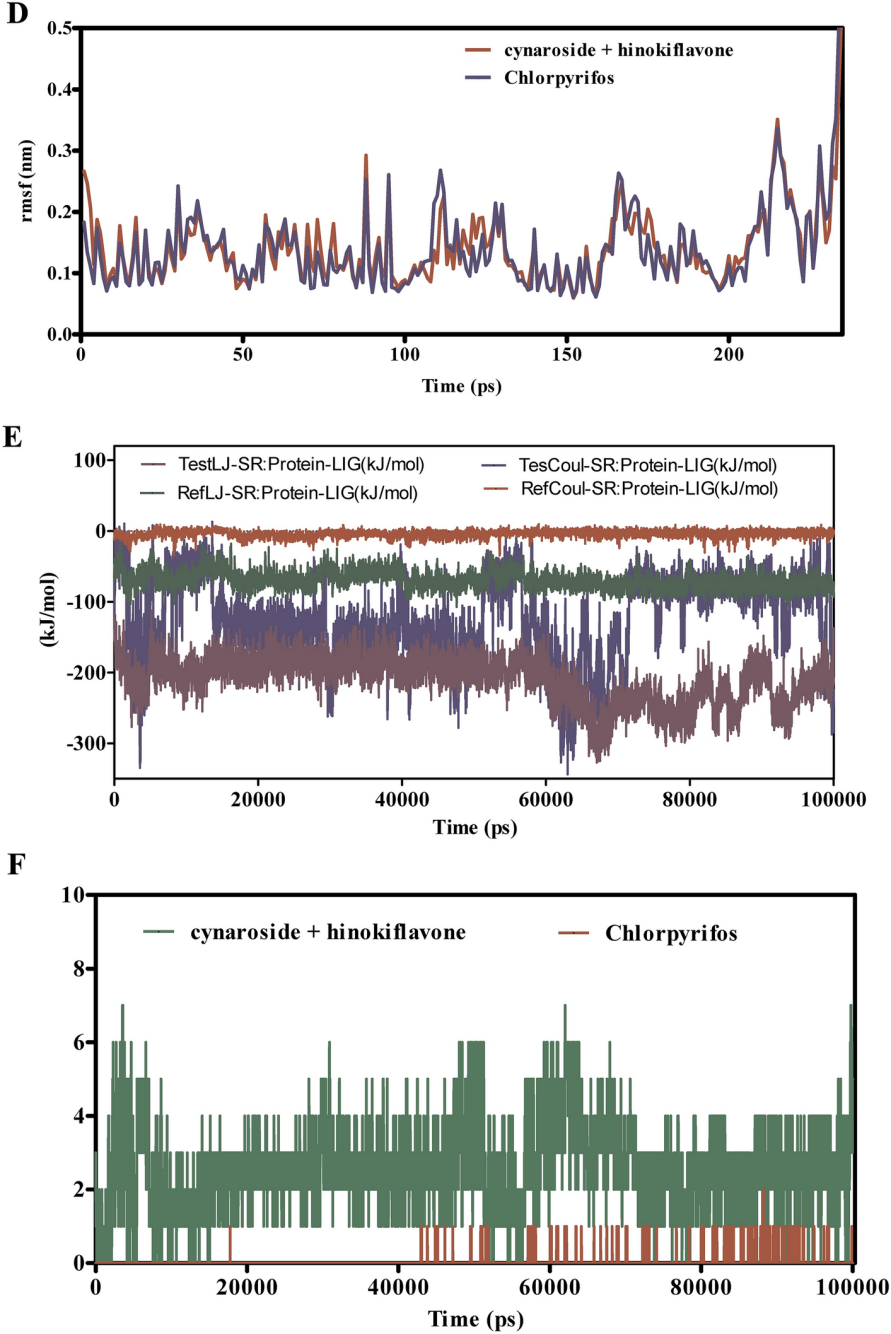
(**A**–**F**) The molecular dynamic simulations of COX1 with ligands- cynaroside + hinkoflavone and chlorpyrifos (reference) for 100 ns. The Root mean square deviation (RMSD), Root mean square fluctuation (RMSF), interaction energies, and Hydrogen bond interactions of ligands- cynaroside + hinkoflavone and chlorpyrifos (reference) for 100 ns were represented. (**A**) Root mean square deviation (RMSD) of ligands- cymaroside + hinkoflavone and chlorpyrifos (reference) for 100 ns. (**B**) Root mean square deviation (RMSD) of COX1 interaction with cymaroside + hinkoflavone and chlorpyrifos (reference) for 100 ns. (**C**) Root mean square fluctuation (RMSF) of ligands cymaroside + hinkoflavone and chlorpyrifos (reference) interaction with receptor for 100 ns. (**D**) Root mean square fluctuation (RMSF) of COX1 interaction with cynaroside + hinkoflavone and chlorpyrifos (reference) for 100 ns. (**E**) Lennard–Jones interactions and Coulomb interactions energies of COX1 receptor and ligands- cynaroside + hinkoflavone and chlorpyrifos (reference) for 100 ns. (**F**) Hydrogen bond interactions of cynaroside + hinkoflavone and chlorpyrifos (reference) with COX1 receptor for 100 ns.

Figure 10A–F examines the interactions of cymaroside + amentoflavone versus chlorpyrifos with COX1 over a 100 ns simulation period. The RMSD profiles in Fig. 10A,B indicate that cymaroside + amentoflavone maintains stable deviation values between 2.5 to 3.2 Å, suggesting consistent binding. In contrast, chlorpyrifos shows higher RMSD fluctuations ranging from 3.0 to 4.5 Å, indicative of a less stable binding configuration within the enzyme’s active site. Figure 10C demonstrates that residues within the binding site interacting with cymaroside + amentoflavone exhibit RMSF values between 1.2 to 1.8 Å, reflecting reduced flexibility and a more rigid binding conformation. Conversely, chlorpyrifos-bound complexes display higher RMSF values (1.5 to 2.5 Å), indicating increased residue mobility and potentially destabilizing interactions. Interaction energy analysis in Fig. 10E shows that cymaroside + amentoflavone forms stronger Lennard–Jones and Coulombic interactions (−150 to −180 kJ/mol) compared to chlorpyrifos (−100 to −130 kJ/mol), suggesting a more energetically favourable and stable binding. Figure 10F reveals that cymaroside + amentoflavone consistently forms 2 to 4 stable hydrogen bonds with distances of 2.8 to 3.2 Å, whereas chlorpyrifos forms fewer and less consistent hydrogen bonds with longer distances (3.2 to 3.6 Å). Cymaroside + amentoflavone demonstrates enhanced binding stability, reduced flexibility, stronger interaction energies, and more stable hydrogen bonding with COX1 compared to chlorpyrifos. These findings suggest that cymaroside + amentoflavone forms a more effective and stable ligand–protein complex.

**Fig. 11:**
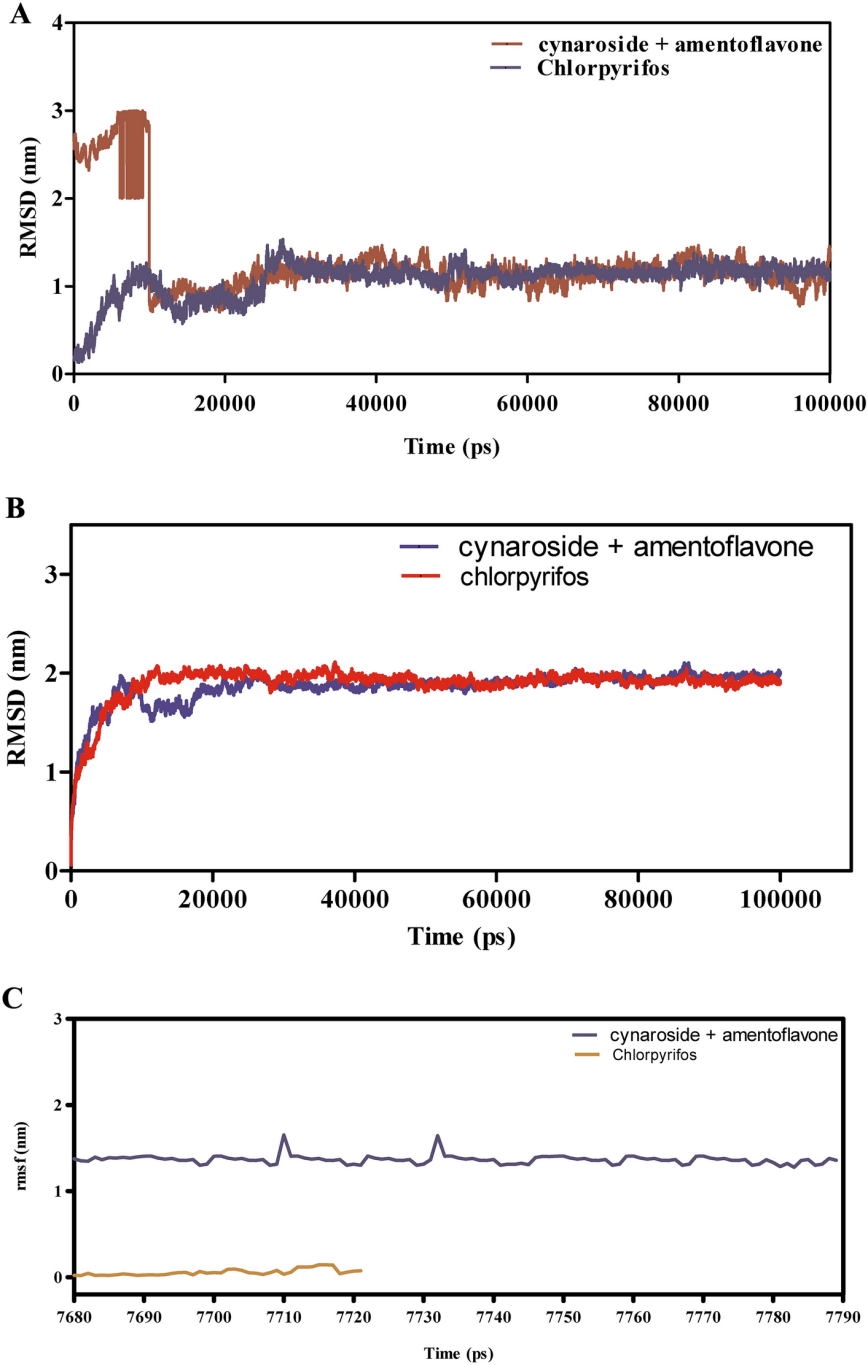

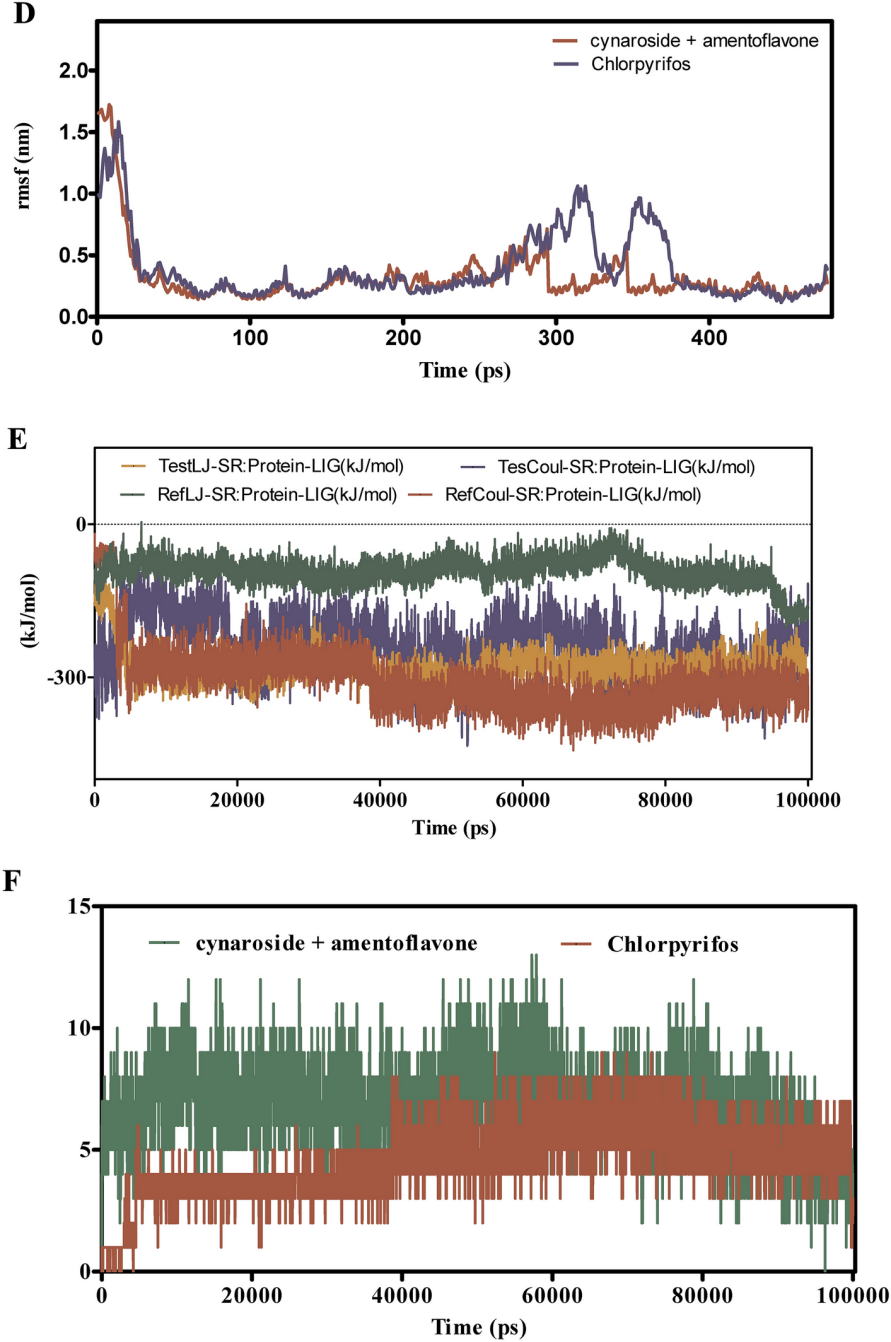
(**A**–**F**) The molecular dynamic simulations of ORs with ligands- cynaroside + hinkoflavone and chlorpyrifos (reference) for 110 ns. The Root mean square deviation (RMSD), Root mean square fluctuation (RMSF), interaction energies, and Hydrogen bond interactions of ligands- cynaroside + hinkoflavone and chlorpyrifos (reference) for 110 ns were represented. (**A**) Root mean square deviation (RMSD) of ligands- cymaroside + amentoflavone and chlorpyrifos (reference) for 110 ns. (**B**) Root mean square deviation (RMSD) of ORs interaction with cymaroside + amentoflavone and chlorpyrifos (reference) for 110 ns. (**C**) Root mean square fluctuation (RMSF) of ligands cymaroside + amentoflavone and chlorpyrifos (reference) interaction with receptor for 110 ns. (**D**) Root mean square fluctuation (RMSF) of ORs interaction with cynaroside + hinkoflavone and chlorpyrifos (reference) for 110 ns. (**E**) Lennard–Jones interactions and Coulomb interactions energies of ORs receptor and ligands- cynaroside + hinkoflavone and chlorpyrifos (reference) for 110 ns. (**F**) Hydrogen bond interactions.

Figure 11A–F presents the MD simulation results for the interactions between cymaroside + amentoflavone and chlorpyrifos with the ORs over a 110 ns trajectory. The RMSD analysis in Fig. 11A indicates that cymaroside + amentoflavone maintains RMSD values fluctuating around 1.5–2.5 Å, reflecting a stable ligand structure. In contrast, chlorpyrifos exhibits higher RMSD deviations, suggesting more pronounced conformational changes. Figure 11B shows that the ORs -ligand complex with cymaroside + amentoflavone maintains a smoother RMSD profile (1–2 Å) compared to chlorpyrifos, which reaches up to 3 Å, indicating weaker binding stability.

Figure 11C,D illustrate that the RMSF values for receptor residues interacting with cymaroside + amentoflavone remain between 0.5–1.5 Å, indicating limited flexibility and a more rigid binding conformation. Conversely, chlorpyrifos induces higher RMSF values exceeding 2 Å in certain regions, suggesting increased local flexibility and potentially less stable binding interactions. Figure 11E shows that cymaroside + amentoflavone forms stronger Lennard–Jones and Coulomb interactions within atomic distances (1–3 Å) compared to chlorpyrifos, which exhibits weaker interaction profiles. Figure 11F highlights that cymaroside + amentoflavone consistently forms more frequent and stable hydrogen bonds within an optimal distance range (1.5–2.5 Å), whereas chlorpyrifos forms fewer and more transient hydrogen bonds with longer distances, indicating weaker interaction stability. Cymaroside + amentoflavone exhibits superior binding stability, reduced receptor flexibility, stronger interaction energies, and more stable hydrogen bonding with the ORs compared to chlorpyrifos. These results suggest that cymaroside + amentoflavone is a more effective and stable ligand for targeting the ORs.

Across all studied proteins: AGI, CEHs, CSPs, COX1, and the Ors, alternative ligand combinations (chamazulene + robustoflavone, cynaroside + hinkoflavone, spathulenol + robustaflavone, and cymaroside + amentoflavone) consistently demonstrate superior binding stability, reduced flexibility, stronger interaction energies, and more stable hydrogen bonding compared to the reference compound chlorpyrifos. These findings collectively highlight the potential of these natural ligand combinations as more effective and stable alternatives to chlorpyrifos in modulating target protein functions. The enhanced binding characteristics of the alternative ligand combinations suggest their potential as viable substitutes or complementary agents to chlorpyrifos, particularly in applications requiring stable and specific protein interactions. Future studies should focus on experimental validation of these computational findings, exploring the biological efficacy and safety profiles of these ligands in relevant biological systems. Additionally, further optimization of these ligand structures could lead to the development of highly potent inhibitors with minimal adverse effects, contributing to safer and more effective therapeutic strategies.

## Conclusion

The present study provides a thorough analysis of the binding efficacy of natural compounds from lemongrass and cedarwood essential oils against key proteins in *Spodoptera litura*. Using homology modeling, energy minimization, and protein-ligand docking, compounds like cynaroside, amentoflavone, and chamazulene displayed strong inhibitory potential, with high binding affinities to COX1, AGI, and ORs. These compounds effectively disrupt critical physiological pathways in the pest, suggesting their potential as eco-friendly, sustainable bioinsecticides. Compared to chlorpyrifos, lemongrass and cedarwood compounds exhibited stronger, more stable interactions, including enhanced hydrogen bonding and hydrophobic interactions, indicating greater binding stability. Additionally, combinations of the best ligands showed synergistic effects, improving binding stability and targeting multiple pathways. The molecular dynamics simulations reinforced these findings, demonstrating superior binding stability, stronger interaction energies, and consistent RMSD and RMSF values for the natural ligands. These results position lemongrass and cedarwood essential oils as promising alternatives to conventional chemical insecticides, with the potential for safer, more effective pest management. Further experimental validation is needed to confirm their biological efficacy and environmental safety.

## Data Availability

All data generated or analysed during this study are included in the manuscript. Once the publication was accepted the data can be access through online manuscript.

## Abbreviations

COX1: Cytochrome c oxidase subunit 1
AGI: Alpha-glucosidase
ORs: Octopamine receptor
CEHs: Carboxylic ester hydrolase
CSPs: Chemosensory proteins
